# Controversies Surrounding Segments and Parasegments in Onychophora: Insights from the Expression Patterns of Four “Segment Polarity Genes” in the Peripatopsid *Euperipatoides rowelli*

**DOI:** 10.1371/journal.pone.0114383

**Published:** 2014-12-03

**Authors:** Franziska Anni Franke, Georg Mayer

**Affiliations:** Animal Evolution & Development, Institute of Biology, University of Leipzig, Talstraße 33, D-04103 Leipzig, Germany; Sars International Centre for Marine Molecular Biology, Norway

## Abstract

Arthropods typically show two types of segmentation: the embryonic parasegments and the adult segments that lie out of register with each other. Such a dual nature of body segmentation has not been described from Onychophora, one of the closest arthropod relatives. Hence, it is unclear whether onychophorans have segments, parasegments, or both, and which of these features was present in the last common ancestor of Onychophora and Arthropoda. To address this issue, we analysed the expression patterns of the “segment polarity genes” *engrailed*, *cubitus interruptus*, *wingless* and *hedgehog* in embryos of the onychophoran *Euperipatoides rowelli*. Our data revealed that these genes are expressed in repeated sets with a specific anterior-to-posterior order along the body in embryos of *E. rowelli*. In contrast to arthropods, the expression occurs after the segmental boundaries have formed. Moreover, the initial segmental furrow retains its position within the *engrailed* domain throughout development, whereas no new furrow is formed posterior to this domain. This suggests that no re-segmentation of the embryo occurs in *E. rowelli*. Irrespective of whether or not there is a morphological or genetic manifestation of parasegments in Onychophora, our data clearly show that parasegments, even if present, cannot be regarded as the initial metameric units of the onychophoran embryo, because the expression of key genes that define the parasegmental boundaries in arthropods occurs after the segmental boundaries have formed. This is in contrast to arthropods, in which parasegments rather than segments are the initial metameric units of the embryo. Our data further revealed that the expression patterns of “segment polarity genes” correspond to organogenesis rather than segment formation. This is in line with the concept of segmentation as a result of concerted evolution of individual periodic structures rather than with the interpretation of ‘segments’ as holistic units.

## Introduction

Arthropods, including spiders, centipedes, crustaceans, insects and allies, are the most diverse and abundant animals on Earth [1], [2]. The evolutionary success of these animals might be attributed to a modular, segmented body design [3], [4]. During embryonic development, segment formation is governed by the hierarchical expression of the so-called “segmentation genes”, which in addition to playing other, pleiotropic roles in the embryo provide positional information for segmental patterning [3], [5]. However, segmentation in arthropods is more than just a simple repetition of metameric units along the body, as arthropods in fact show two types of segmentation: the embryonic parasegments, and the adult segments. The parasegments are the initial metameric units of the embryo, but due to a re-segmentation are replaced by the definitive segments that lie out of register with parasegments [6], [7].

The definitive segments comprise typical metameric units of adult arthropods, which are seen at least in those body parts that have retained the ancestral, homonomous architecture, including the trunk of centipedes and woodlice, the metasoma of scorpions, and the abdomen of insects. In these body regions, the segments are demarcated by the anterior and posterior borders of sclerites and contain additional segmental structures, such as tracheal openings, gills, ostia of the heart, and limbs with associated muscles [3], . In other body parts, the segments have fused to distinct tagmata and, thus, are no longer recognisable as individual units, for instance in the head and thorax of insects or the prosoma and opisthosoma of spiders [3], [4], [11], [12].

In contrast to the adult segments, the embryonic parasegments occur early in development and are not retained in adults. They are regarded as true metameric compartments [13], [14] and their boundaries are generated by an interaction of the canonical Hedgehog and Wnt/Wingless signalling pathways and the transcription factor Engrailed, which are expressed in cell rows flanking the parasegmental boundary [3], [7], . The expression of *wingless* occurs anterior to this boundary, while *hedgehog* and *engrailed* are expressed posterior to it.

At least in some chelicerate [16], crustacean [19] and insect [14], [20], [21] embryos, the parasegmental boundary is evidenced by a transverse groove between the *wingless* and *engrailed* domains. This groove, however, disappears during the re-segmentation of the embryo, after which a new (segmental) boundary arises posterior to the *hedgehog* and *engrailed* domains. This boundary corresponds to the border between adjacent sclerites in adult arthropods [5], [9]. Due to the re-segmentation of the embryo, segments and parasegments show an entirely different spatial relationship to the expression patterns of the four commonly studied “segment polarity genes”; while *engrailed* and *hedgehog* are expressed anteriorly and *wingless* and *cubitus interruptus* posteriorly in each parasegment, the opposite occurs in the definitive segments [16], .

Based on the similarities in the expression patterns of these genes, the embryonic parasegments of arthropods have been homologised with the segments of annelids [24], [25]. For example, despite apparent deviations in some species [26]–[28] the anterior-to-posterior sequence of expression of homologs of these genes is basically the same in annelids and arthropods [24]. Therefore, it has been assumed that the last common ancestor of protostomes was segmented and that the metameric exoskeleton of arthropods has evolved out of phase with this ancestral segmentation; however, the ancestral segmentation pattern is still retained in the arthropod embryo [24]. According to this scenario, one would expect that parasegments (or their vestiges) also occur as initial metameric units in embryos of one of the closest arthropod relatives, the Onychophora (velvet worms) [24], [29].

**Figure 1:**
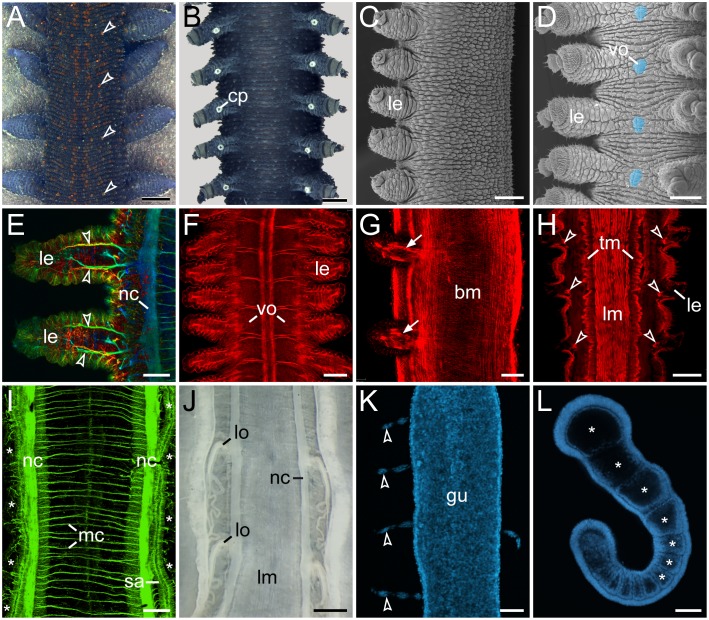
Segmental and non-segmental features in Onychophora. (A, B, J) Light micrographs. (C, D) Scanning electron micrographs. (E–I, K, L) Confocal micrographs. (A) Trunk of a specimen of *Euperipatoides rowelli* in dorsal view. Note the segmentally repeated, terracotta-coloured papillae. (B) Trunk of a male *E. rowelli* in ventral view illustrating segmentally arranged crural papillae. (C) Trunk of a juvenile of *Metaperipatus inae* in lateral view. Note that segmentation is not evident in the integument. (D) Trunk of a juvenile of *M. inae* in ventral view. Segmentally arranged ventral/preventral organs between each leg pair are highlighted artificially in light-blue. (E) Legs of an embryo of *Principapillatus hitoyensis* in dorsal view. Anti-acetylated alpha-tubulin immunolabelling. Note the segmental arrangement of the anterior and posterior leg nerves (arrowheads). (F) Segmentally repeated anlagen of the ventral and preventral organs in an embryo of *Epiperipatus biolleyi* in ventral view. Phalloidin-rhodamine labelling of f-actin. (G) Segmental limb muscles (arrows) in an embryo of *E. rowelli* in lateral view. Phalloidin-rhodamine labelling. (H) Segmental arrangement of intrinsic leg muscles (arrowheads) in *E. rowelli*. Horizontal Vibratome section, phalloidin-rhodamine labelling. Note that the longitudinal and transverse musculature does not show any segmentation. (I) Ventral body wall of an embryo of *P. hitoyensis* in dorsal view. Anti-acetylated alpha-tubulin immunolabelling. Asterisks indicate the position of legs. Note that the median commissures are not arranged in a segmental fashion. (J) Enlarged modified nephridia ( = labyrinth organs) in the fourth and fifth leg-bearing segments. Note their segmental arrangement. Note also the lack of segmental ganglia associated with nerve cords. (K) Dissected midgut of a fully developed embryo of *P. hitoyensis* with segmentally arranged cellular strands (cf. [47]). DNA staining with Hoechst. (L) Embryo of *P. hitoyensis* in lateral view showing the segmental somites (coelomic cavities marked by asterisks). Subset of a confocal z-series showing an optical sagittal section. Abbreviations: *bm*, musculature of the body wall; *cp*, crural papilla; *gu*, midgut; *le*, leg; *lm*, longitudinal musculature; *lo*, labyrinth organ; *mc*, median commissures; *nc*, nerve cord; *sa*, salivary gland; *tm*, transverse musculature; *vo*, ventral/preventral organs. Scale bars: 1 mm (A), 500 µm (B), 300 µm (C–D), 50 µm (E), 100 µm (F, G, I, K, L), 200 µm (H), 1 mm (J).

The onychophoran body exhibits a mixture of segmental and non-segmental features [30],[31]. While various structures, including limbs, crural papillae, ventral and preventral organs, cellular strands associated with midgut, nephridia, and embryonic somites ( = coelomic cavities), clearly show a metameric arrangement, no segmental organisation is evident in the cuticle or longitudinal musculature (Figure 1A–L; [30], ). Therefore, in contrast to arthropods, there are no clear segmental boundaries in adult onychophorans [8], . Consequently, the only segmental structures that might be homologous in Onychophora and Arthropoda are the limbs, the motor neurons supplying these limbs, and the nephridia and their derivatives [36]–[38].

Irrespective of whether or not they are homologous, various segmental structures might use a similar genetic scaffold in onychophorans and arthropods for metameric positioning along the body. Gene expression studies on embryos of the onychophoran *Euperipatoides kanangrensis* [29], [39] indeed revealed that the genes *engrailed*, *cubitus interruptus*, *hedgehog* and *wingless* are expressed in the same anterior-to-posterior order as in arthropods. However, based on the data available, it is impossible to determine whether or not onychophorans have true parasegmental boundaries. On the one hand, the expression of *engrailed* and *wingless* is graded and there is no precise cellular boundary between their domains at least within the trunk [29], which speaks against the existence of a parasegmental boundary in Onychophora. On the other hand, the *engrailed* domain extends beyond the segmental furrow [29], which would thus correspond neither to the segmental nor to the parasegmental boundary. Thus, the existence of the parasegmental boundary in the onychophoran embryo remains ambiguous.

To determine whether or not the transverse furrows retain their position within the *engrailed* domain throughout development, or whether a new segmental furrow arises posterior to the *engrailed* domain, we analysed the spatiotemporal relationship of transverse furrows and other segmental structures with respect to the *engrailed* domains in embryos of the onychophoran *Euperipatoides rowelli*. Our study covers more developmental stages than analysed before and provides a more complete picture of the anatomical changes throughout development. To further clarify whether segments or parasegments are the initial metameric units, we analysed the expression patterns of three additional genes, including *cubitus interruptus*, *wingless* and *hedgehog*, that are known to be involved in the developmental control of segment polarity in embryos of *Drosophila melanogaster* [7], [40], [41]. Furthermore, we examined in detail the spatial relationship of the expression patterns of these four genes to the individual metameric structures, such as limb buds and the anlagen of the ventral and preventral organs [30], [42], to determine whether the segmented body organisation of Onychophora is compatible with the interpretation of segments as holistic units [43]–[45] or rather with the concept of segmentation as a result of concerted evolution of individual periodic structures [8], [10], [46].

## Materials and Methods

### Specimen collection

Specimens of *Euperipatoides rowelli* Reid, 1996 (Onychophora, Peripatopsidae) were collected from rotted logs in the Tallaganda State Forest (New South Wales, Australia) in October 2010 and 2011 and maintained in the laboratory as described previously [47]. The necessary permits for the collection of onychophorans were obtained from the Forestry Commission of New South Wales, Australia (Special Purposes Permit for Research no. XX51212). We have chosen *E. rowelli* because this species is highly abundant and can be collected easily outside national parks [48]–[50]. Moreover, *E. rowelli* has become the most studied onychophoran species to date [51], the biology, anatomy, development, phylogeny and population genetics of which have been analysed extensively [30], [31], [37], [38], [42], [47]–[50], . Currently, the genome of *E. rowelli* is being sequenced (http://www.hgsc.bcm.tmc.edu/content/i5k-velvet-worm), which will provide additional resources for working with this “model” onychophoran species.

### Dissection and fixation of embryos

For cytochemical and gene expression studies, females of *E. rowelli* were anaesthetised in chloroform vapour and the reproductive tracts dissected and transferred into dishes containing a physiological saline [71]. After dissecting the embryos from the uteri, the embryonic membranes were removed manually using two forceps. The embryos were then fixed overnight in 4% paraformaldehyde in phosphate-buffered saline (PBS; 0.1 mol/L, pH 7.4) and staged according to Walker and Tait [72] with the following modification. We classified stage V embryos in a more restrictive way by using the following features: (i) cerebral grooves ( = anlagen of the hypocerebral organs) appear as longitudinal slits in the middle of each cephalic lobe, and (ii) the anlagen of the last (15^th^) pair of walking legs have formed. After staging, the embryos were either processed further for cytochemical experiments or dehydrated in a graded methanol series and stored at −20°C for subsequent gene expression experiments.

### Cytochemistry

For cytochemical studies, the embryos fixed in 4% paraformaldehyde were rinsed several times in PBS and incubated overnight at room temperature in a solution containing the f-actin marker phalloidin-rhodamine (Invitrogen, Carlsbad, CA) as described previously [30]. After repeated rinses in PBS, the embryos were counterstained with the DNA-selective fluorescent dye Bisbenzimide (H33258; Sigma-Aldrich, St. Louis, MO, USA; 1 µg/mL in PBS) and rinsed again in PBS. The embryos were then mounted between two coverslips in Vectashield Mounting Medium (Vector Laboratories, Burlingame, CA) and analysed with the confocal laser-scanning microscope Zeiss LSM 510 META (Carl Zeiss MicroImaging GmbH, Jena, Germany).

### Identification and amplification of gene fragments

Library preparation and assembly of the embryonic transcriptomes from *E. rowelli* were performed as described previously [58]. Local tBLASTn searches [73] were conducted using transcriptome libraries from different embryonic stages [58]. Previously published sequences from other onychophoran and arthropod species were used as queries [29], [74], [75]. RNA was isolated from pooled embryos of different developmental stages using TRIzol Reagent (Invitrogen) and RNeasy MinElute Cleanup Kit (Qiagen, Hilden, Germany) according to the manufacturers’ protocols. First-strand synthesis was performed using random hexamer primers and Superscript III polymerase (Invitrogen). Second-strand synthesis was carried out with DNA Pol I polymerase (Invitrogen). The obtained cDNA was purified using NucleoSpin Extract II-Kit (Macherey-Nagel, Düren, Germany) following the manufacturer's protocol. Fragments of *engrailed*, *cubitus interruptus, wingless* and *hedgehog* were amplified using specific primers (Table 1). The corresponding sequences were made available under the GenBank accession numbers KF218600–KF218603.

**Table 1:** Specific primers used for PCR.

Gene	Fragmentlength (in bases)	Direction	Primer sequence
*engrailed*	668	forward	CTGAACTGGGTCGATCTGAATATCTCG
		reverse	CGTATATTTGCCTCGCTTACAAG
*cubitus* *interruptus*	602	forward	TCCTCGCCCGTTCTGCCACT
		reverse	TCCAGGCAGTTCACGCCGTT
*wingless*	1019	forward	TCCGTGCCGCGTACTCTACCT
		reverse	CCTCACTTTTATAACCTCTACCACA
*hedgehog*	607	forward	TCACAGGGGCAAAAGCCCAGT
		reverse	CGATTGGCGGTTGAGGCTGGT

### Sequence alignment and phylogenetic analyses

The identified sequences of *E. rowelli* homologs of *engrailed* (*Er-en*), *cubitus interruptus* (*Er-ci*), *wingless* (*Er-wg*) and *hedgehog* (*Er-hh*) were compared to the sequences available from the NCBI database using BLAST searches. The corresponding amino acid sequences from *E. rowelli* were analysed together with those from several other metazoan species, including the closely related onychophoran species *E. kanangrensis* (see Table S1). Sequence alignments (481, 200, 572 and 429 amino acid positions for *engrailed*, *cubitus interruptus*, *hedgehog* and *wingless*, respectively) were generated with the online version of MAFFT [76] using the FFT-NS-i strategy (see Figure S1). The appropriate models for protein evolution (LG+G+F for *engrailed* DAYHOFF+I+G for *cubitus interruptus*, and LG+I+G for *hedgehog* and *wingless*) were selected using ProtTest 3.2 [77] according to the Akaike Information Criterion (AIC) [78]. Maximum Likelihood analyses were performed using Pthread-based version of RAxML v7.2.8 [79]. Nodal support was calculated using 100 bootstrap replicates. Phylogenetic trees were visualised with iTol [80] and edited with Adobe (San Jose, CA, USA) Illustrator CS5.1.

### Molecular cloning, probe preparation and whole-mount *in* *situ* hybridization

Gene fragments were cloned into the pGEM-T Vector System I (Promega Corporation, Madison, WI, USA). Digoxigenin- and biotin-labelled RNA probes were prepared using DIG RNA Labeling Kit SP6/T7 and Biotin RNA Labeling Mix (Roche, Mannheim, Germany). Whole-mount *in* *situ* hybridization was performed as described previously [22], [81] with the following modifications. The embryos stored in 100% methanol were rehydrated in a graded methanol series (2**×**100%, 75%, 50% and 25% in PBST [PBS+0.1% Tween-20], 7 min each). Pre-hybridization (six hours) and hybridization steps (three days) were carried out at 60°C. 100–500 ng of the probes were diluted in 500 µl hybridization solution (50% formamide, 5xSSC, 50 µg/mL heparin, 50 µg/mL yeast tRNA, 5% Dextran sulphate, 0.1% Tween-20). Post-hybridization washes included several rinses in hybridization buffer at 60°C, followed by several rinses in a washing solution (2xSSC+0.1% Tween-20) at 60°C and in PBST at room temperature. The embryos were then incubated for 3 hours in a blocking solution (10% normal goat serum in PBST) at room temperature, followed by an incubation with anti-digoxigenin alkaline phosphatase-conjugated antibody (Roche), diluted 1∶1000 in blocking solution for two days at 4°C. After several washes with PBST at room temperature, NBT/BCIP staining solution (Roth, Karlsruhe, Germany) was added. The reaction was stopped after the desired staining was achieved by several washes with PBST. Double whole-mount *in situ* hybridization was carried out as described by Schinko et al. [82] with the following modifications. After detection of the first colour, the embryos were incubated in inactivation buffer (50% formamide, 5xSSC, 0.1% Tween-20, 10% sodium dodecyl sulphate) at 60°C in a heating block. Embryos were then washed in blocking solution for two hours and the antibody for the second staining was added at a dilution of 1∶100 in blocking solution. The second colour reaction was stopped by several washes with PBST and the embryos were then re-fixed in 4% paraformaldehyde and stored at 4°C. For nuclear staining, the DNA-selective fluorescent dye SYBR Green (Invitrogen) was applied according to the manufacturer’s protocol.

### Microscopy and image processing

The embryos were analysed under a stereomicroscope (Leica WILD M10 with a WILD MDG 17 Stand; Leica Microsystems, Wetzlar, Germany) and a transmitted-light microscope (Leica Leitz DMR; Leica Microsystems) equipped with a colour digital camera (PCO AG SensiCam, Kelheim, Germany). Several micrographs were taken from each embryo at different focal planes and merged to a single image using the Auto-Blend Layers function in Adobe Photoshop CS5.1. Brightness and contrast were adjusted using Photoshop CS5.1. Final panels and diagrams were designed with Illustrator CS5.1 and exported to Tagged Image File Format files. Confocal laser-scanning microscopy and image processing were performed as described previously [30].

## Results

### Developmental origin and fate of transverse segmental furrows in embryos of *E. rowelli*

**Figure 2:**
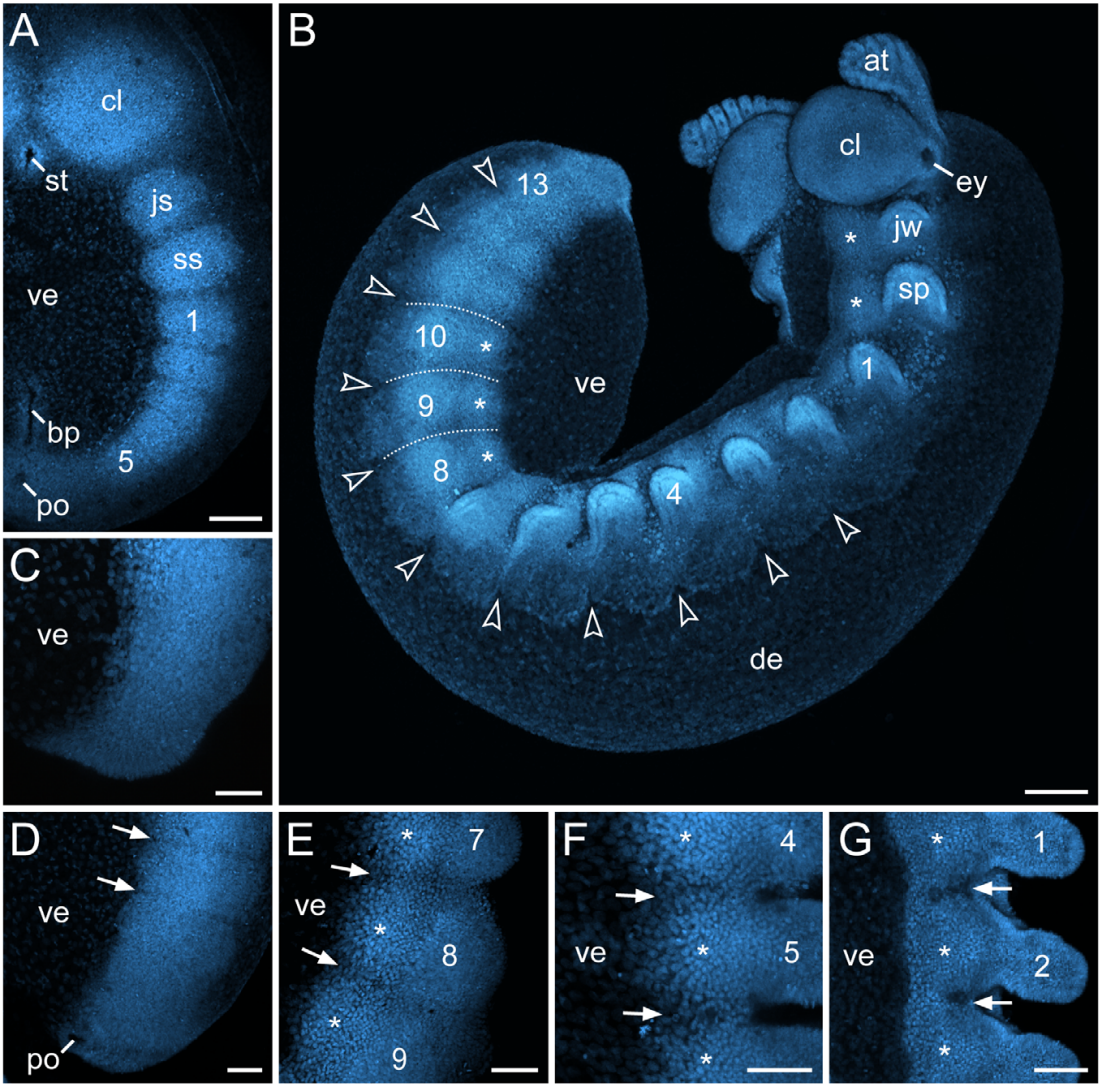
Developmental origin and fate of transverse segmental furrows in embryos of *E. rowelli* at successive developmental stages. Confocal micrographs of embryos labelled with Bisbenzimide. Leg-bearing segments and corresponding limbs are numbered. Asterisks indicate the anlagen of the ventral and preventral organs. (A) Stage I embryo in ventral view. Note distinct borders between the developing segments. (B) Late stage III embryo in ventro-lateral view. Arrowheads point to the dorsal indentations in the ectoderm. Dotted lines demarcate the transverse furrows. (C–G) Chronological sequence of the formation of the segmental furrows. Embryos in lateral (C–E) and ventral view (F, G). Arrows point to the segmental furrows. (C) Posterior end of a stage II embryo. Note that furrows are not visible yet. (D) Posterior end of a stage III embryo. (E) Midbody of an early stage IV embryo. Note an additional longitudinal furrow separating the anlagen of the ventral/preventral organs and the developing limbs. (F) Anterior body region of a stage IV embryo. (G) Anterior body region of a stage V embryo. Abbreviations: *at*, developing antenna; *bp*, blastoporus; *cl*, cephalic lobe; *de*, dorsal extra-embryonic tissue; *ey*, eye; *js*, jaw segment; *jw*, jaw; *st*, stomodaeum; *sp*, slime papilla; *ss*, slime papilla segment; *po*, proctodaeum; *ve*, ventral extra-embryonic tissue. Scale bars: 200 µm (A, B), 100 µm (C–G).

The initial segmental structures appearing in the embryo of *E. rowelli* are the paired mesodermal somites ( = coelomic cavities; Figure 2A; see also Figure S2). Due to an anterior-to-posterior progression in development (i.e., the anterior segments are further advanced than the posterior ones), the somites arise sequentially in the antennal, jaw and slime papilla segments, followed by the trunk segments (Figure 2A). As soon as the mesodermal somites have formed, transverse furrows appear at regular intervals in the overlying ectoderm (Figure 2A–D; see Figure S2). Although the formation of these furrows lags behind that of somites, their position along the body corresponds exactly to the border between each adjacent somite (see Figure S2).

During further development, paired segmental limb buds (numbered in Figure 2B, E–G) arise in an anterior-to-posterior progression from lateral portions of the germ band. Simultaneously, the median portions of the germ band give rise to segmental thickenings (asterisks in Figure 2B, E–G) that are the anlagen of the ventral and preventral organs (see ref. [42] for details on the developmental fate of these thickenings). Shortly after the limb buds and the anlagen of the ventral and preventral organs have formed, the lateral ectodermal portions of the germ band extend dorsally in a regular, undulating fashion. This regular growth gives rise to conspicuous segmental indentations in the ectoderm (arrowheads in Figure 2B), which correspond in position to the transverse furrows that separate the segmental anlagen of limbs and the ventral/preventral organs along the body (dotted lines in Figure 2B and arrows in Figure 2E–G). These indentations show that there is a defined structure in the ectoderm and that the furrows are not simple undulations caused by the bulging, underlying mesodermal somites.

Following the anterior-to-posterior progression in development, the transverse segmental furrows and the dorsal indentations of the ectoderm become less prominent at the anterior end in stage IV embryos and are hardly detectable at stage V (Figure 2B, F, G). Neither the transverse furrows nor the segmental indentations persist beyond this developmental stage in *E. rowelli* (cf. [30], [42], [56], [62]).

### Identification of homologs *of engrailed, cubitus interruptus, wingless* and *hedgehog* in *E. rowelli* and phylogenetic analyses

Irrespective of the assembly filters used (F15, F25, and F30; see ref. [58] for details on methodology), we identified contigs of only one homolog of each gene (*Er-en, Er-ci, Er-hh* and *Er-wg*) in our transcriptomic data. However, since the complete genome sequence is unavailable for Onychophora, we cannot rule out the possibility that there might be additional copies of these genes in the genome, although these might not be expressed during development. To determine whether the identified homologs from the onychophoran *E. rowelli* are orthologous to the corresponding sequences from arthropods, we carried out phylogenetic analyses (see Table S1 and Figure S3). In the resulting cladograms, the identified homologs *Er-en, Er-hh*, *Er-ci* and *Er-wg* form sister groups to the corresponding sequences from *E. kanangrensis* (see Figure S3). These results confirm that the identified sequences of *E. rowelli* are indeed orthologs rather than paralogs of the corresponding arthropod genes.

### Expression of *engrailed* during development in *E. rowelli*

**Figure 3:**
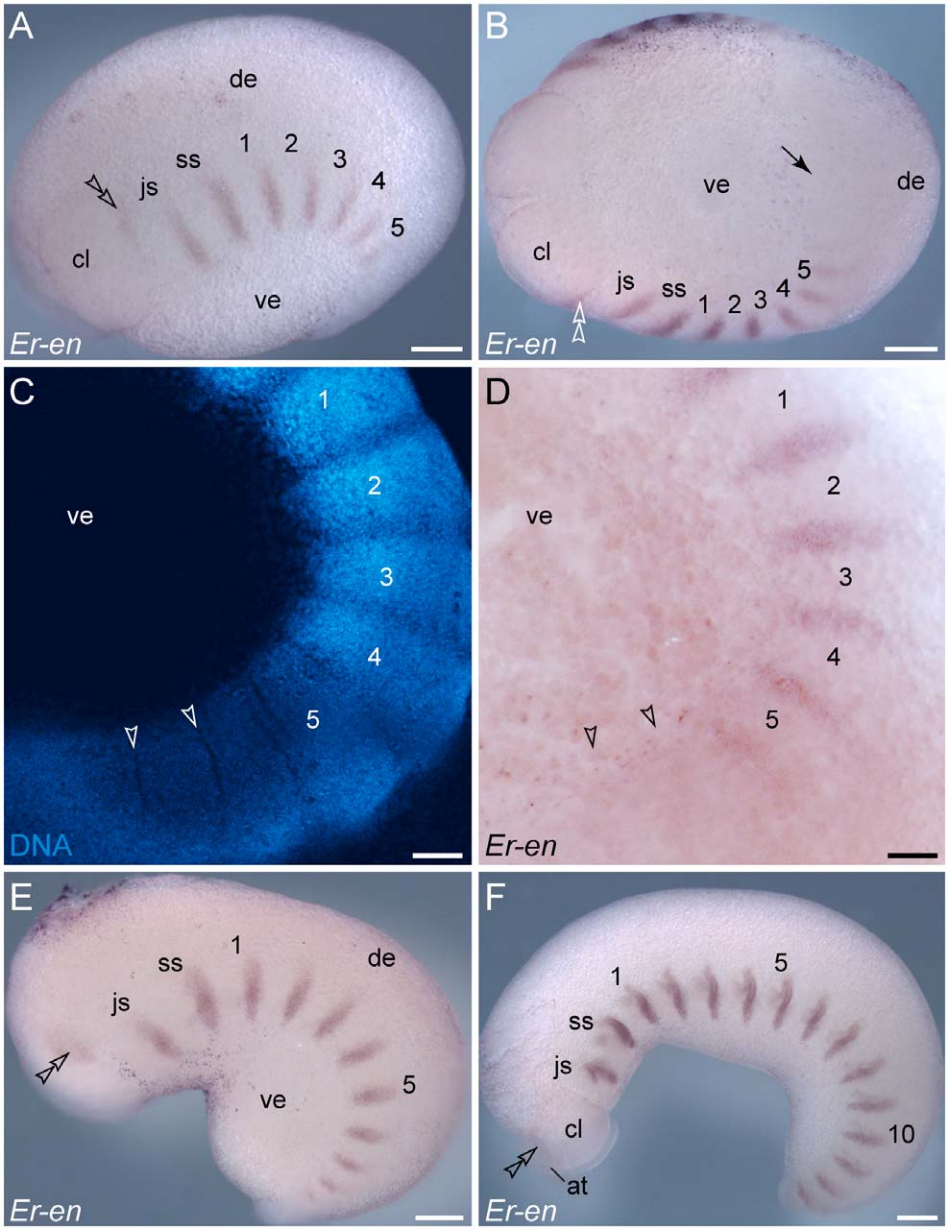
Expression of *engrailed* in embryos of the onychophoran *E. rowelli* at subsequent developmental stages. Anterior is left in A, B, E, F and up in C, D. Leg-bearing segments and corresponding limbs are numbered. Note the repeated stripes along the body and the lack of expression in the dorsal and ventral extra-embryonic tissue. Double-arrowheads in A, B, E and F indicate the dorsally located domain of *engrailed* in the cephalic lobe. Arrowheads in C and D point to the transverse ectodermal furrows, which lack *engrailed* expression. (A) Early stage II embryo in lateral view. (B) The same embryo as in A in ventral view. Note the lack of expression in the posterior region and around the proctodaeum (black arrow). (C) Confocal micrograph of the posterior end of the same embryo as in A labelled with the DNA marker SYBR Green. (D) Light micrograph of the same embryo as in C. (E) Early stage III embryo in lateral view with eleven *engrailed* stripes along the body and a spot-shaped domain in the developing antenna (arrowhead). (F) Late stage III embryo in lateral view with 15 *engrailed* stripes along the body (13 within the trunk) and an additional domain in the developing antenna (arrowhead). Abbreviations: *at*, developing antenna; *cl*, cephalic lobe; *de*, dorsal extra-embryonic tissue; *js*, jaw segment; *ss*, slime papilla segment; *ve*, ventral extra-embryonic tissue. Scale bars: 250 µm (A, B, E, F), 100 µm (C, D).

**Figure 4:**
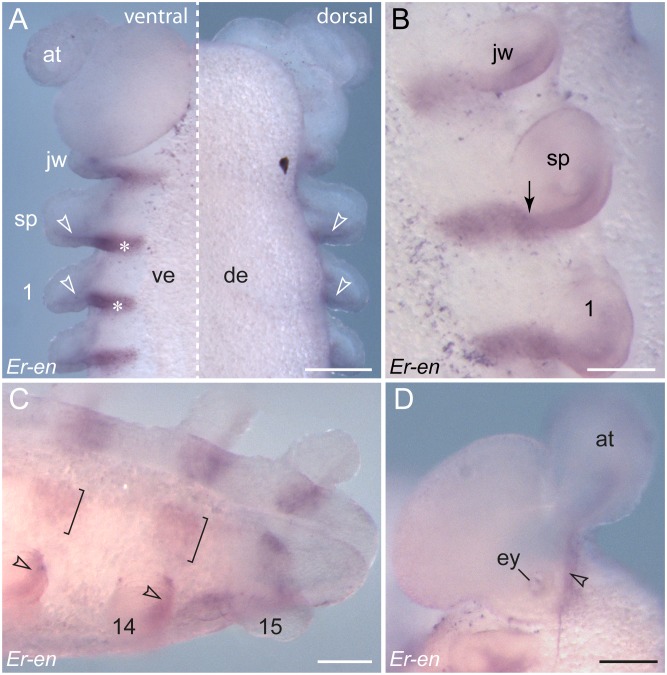
Details of *engrailed* expression in embryos of *E. rowelli*. Leg-bearing segments and corresponding limbs are numbered. (A) Ventral (left) and dorsal perspective (right) of the anterior end of a stage IV embryo. Note the expression in the ventral ectoderm (asterisks) and in the ectoderm and mesoderm of each developing limb (arrowheads). (B) Details of the anlagen of a jaw, a slime papilla and the first leg in a stage IV embryo. Arrow points to the transition between a wide ventral stripe and a narrow domain within the limb, which become separate during further development. (C) Ventro-lateral view of the posterior end of a stage V embryo. Note that the domains within the limb buds (arrowheads) and those on the ventral body surface (square brackets) have become separate. The latter correspond to the anlagen of the ventral and preventral organs [30], [42], [56]. (D) Lateral view of a developing antenna in a stage IV embryo. Abbreviations: *at*, developing antenna; *de*, dorsal extra-embryonic tissue; *ey*, developing eye; *jw*, embryonic jaw; *sp*, embryonic slime papilla; *ve*, ventral extra-embryonic tissue. Scale bars: 250 µm (A), 125 µm (B–D).

During embryogenesis of *E. rowelli*, *engrailed* is expressed in segmentally repeated stripes along the germ band, whereas no expression is seen in the ventral and dorsal extra-embryonic tissue (Figures 3A–F, 4A–D). As the embryo grows, *engrailed* stripes are added posteriorly and increase in size towards the anterior end, following the anterior-to-posterior progression along the body (Figure 3A–F). Notably, the stripes occur after the segmental furrows have formed and the signal is graded, as it does not show clear expression boundaries (cf. Figure 3C, D; see ref. [29] for similar data obtained from the closely related species *E. kanangrensis*). In contrast to other segments, the *engrailed* domain is weaker and situated more dorsally in the antennal segment (Figure 3A, B). Shortly after the antenna has formed, the shape of this domain transforms from a stripe to a spot-shaped domain, which follows the elongation of the developing antenna (arrowheads in Figure 3E, F). This results in an elongated domain at the antennal basis, which extends further posteriorly and is still seen above the eye anlage later in development (Figures 3F, 4D).

**Figure 5:**
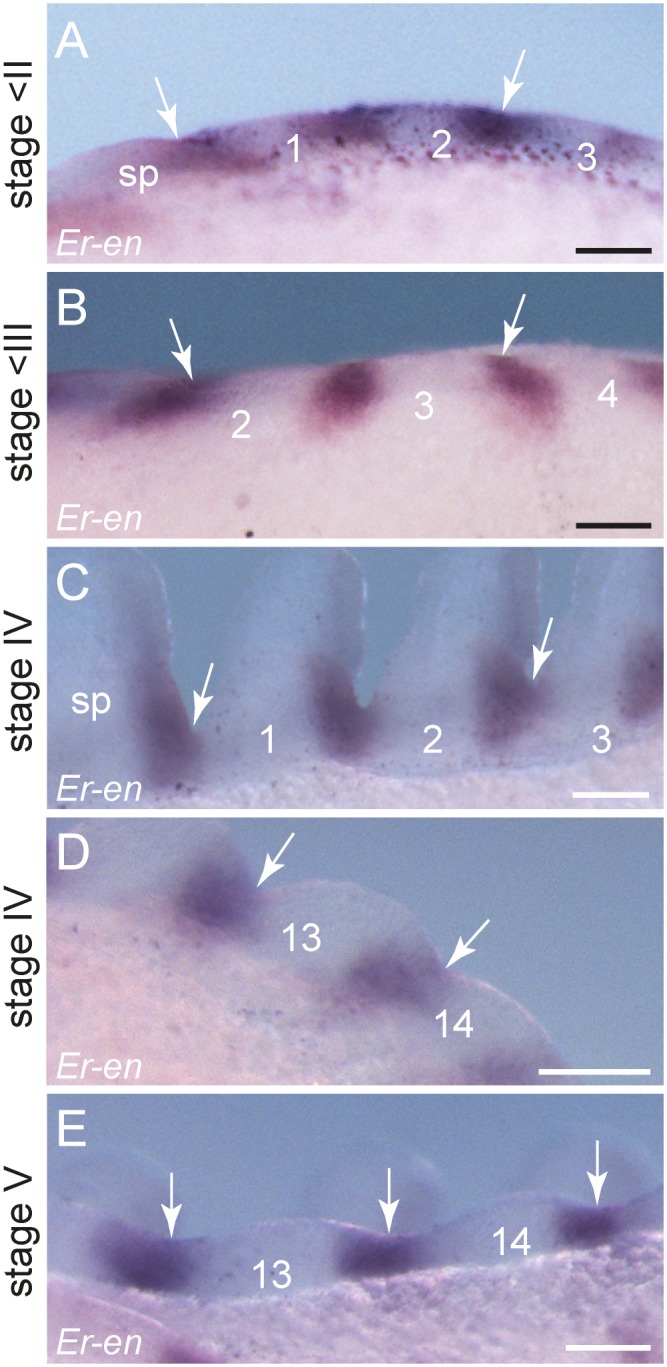
Spatial relationship between segmental furrows and repeated *engrailed* domains in embryos of *E. rowelli*. Anterior is left in all images. Leg-bearing segments and corresponding limbs are numbered. Arrows indicate the position of segmental furrows in embryos of *E. rowelli* at subsequent developmental stages in ventral (A, C, D), dorsal (B), and ventro-lateral perspectives (E). Abbreviation: *sp*, anlage of the slime papilla/slime papilla segment. Scale bars: 100 µm (A, B, D, E,), 125 µm (C).

The expression of the remaining *engrailed* stripes in the embryo precedes the formation of limb buds (Figure 3A). Notably, each *engrailed* stripe extends beyond the segmental furrow in the early embryo (Figure 5A). This pattern persists throughout development and no migration or shift of the initial furrow, or establishment of a new segmental furrow is evident (Figure 5A–E). When the limb buds arise, each *engrailed* stripe follows the curvature of the corresponding limb bud, in which it is located posteriorly in both ectoderm and mesoderm (Figures 3F, 4A–C, 5C–E). While no *engrailed* expression occurs dorsally, each *engrailed* stripe continues from the limb bud to the ventral ectoderm (Figure 4A, B). After all fifteen leg-bearing segments have formed, the initial stripes of *engrailed* are subdivided in two separate domains: a lateral domain associated with the limb bud, and a median domain corresponding to the segmental anlage of the ventral and preventral organs (Figure 4C).

### Expression of *cubitus interruptus* is anterior to each *engrailed* domain in embryos of *E. rowelli*

**Figure 6:**
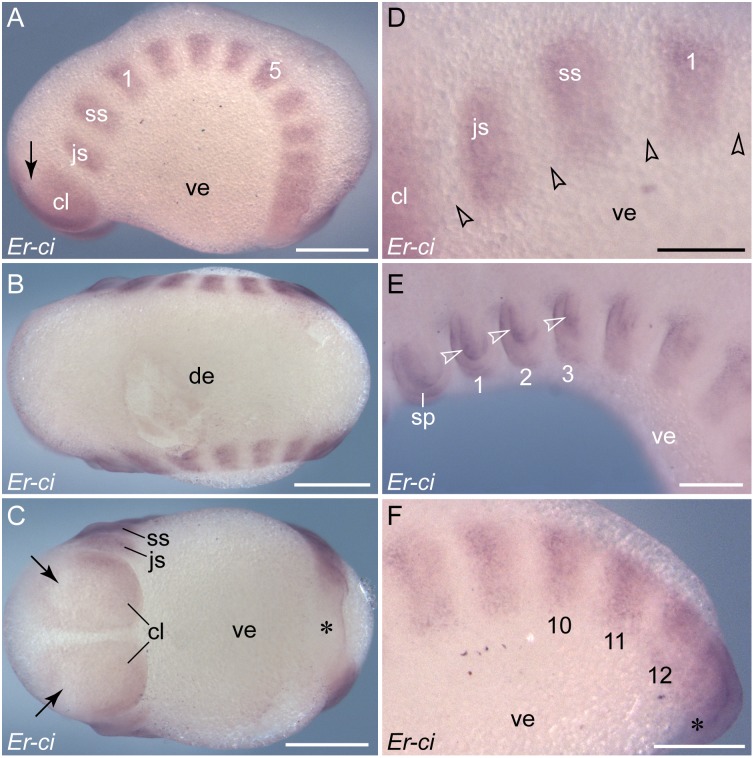
Expression of *cubitus interruptus* in embryos of *E. rowelli*. Anterior is left in all images. Leg-bearing segments and corresponding limbs are numbered. (A–C) Stage II embryo in lateral, dorsal and ventral views. Note the continuous belt of expression on each side of the body at the posterior end, which dissolves into nearly rectangular domains towards the anterior end. Note also the expression-free areas in the cephalic lobes (arrows) and around the proctodaeum (asterisk). (D) Detail of the anterior-most expression domains in a stage II embryo in lateral view. Arrowheads point to wide gaps between the domains. (E) Anterior portion of a stage IV embryo in lateral view showing expression in the ectoderm and mesoderm (somite walls) of the limb anlagen (arrowheads). (F) Posterior end of the same embryo as in E. Note the remnant of the continuous belt of expression near the proctodaeum (asterisk), which dissolves into solitary domains towards the anterior end. Abbreviations: *cl*, cephalic lobe; *de*, dorsal extra-embryonic tissue; *js*, jaw segment; *sp*, embryonic slime papilla; *ss*, slime papilla segment; *ve*, ventral extra-embryonic tissue. Scale bars: 500 µm (A–C), 250 µm (D–F).

**Figure 7:**
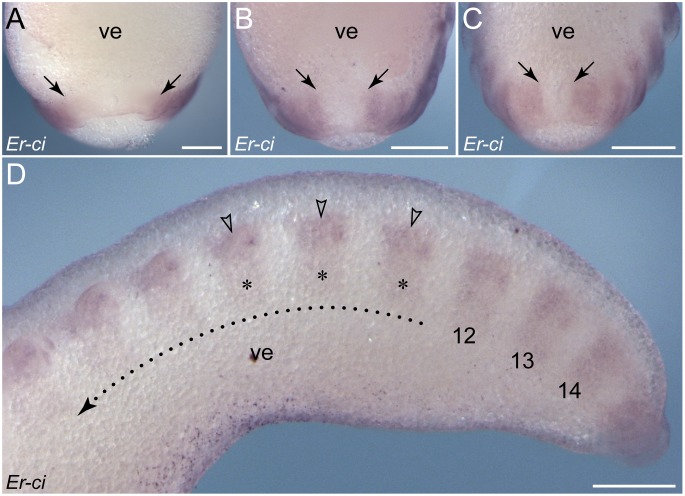
Details of *cubitus interruptus* expression in embryos of *E. rowelli*. (A–C) Posterior ends of embryos at developmental stages II, III and IV in ventral view. Arrows indicate the posterior-most regions of expression, which subsequently move towards the proctodaeum. (D) Posterior end of a stage IV embryo in lateral view. Leg-bearing segments and corresponding limbs are numbered. Dotted line with an arrow indicates the direction of decreasing expression in the ventral ectoderm (asterisks) but increasing expression in the limb anlagen (arrowheads) towards the anterior end. Abbreviation: *ve*, ventral extra-embryonic tissue. Scale bars: 250 µm (A–D).

In contrast to *engrailed*, which is expressed in stripes, *cubitus interruptus* is first expressed as a continuous belt at the posterior end of the embryo, excluding the region around the proctodaeum (Figures 6A–C, F, 7A–C). We were unable to detect *cubitus interruptus* expression in embryos earlier than stage II and, therefore, cannot exclude that this gene might be also expressed in a continuous belt at the anterior end. The continuous belt of expression persists at the posterior end, whereas its anterior part dissociates into increasingly well-defined, segmental, rectangular domains (Figures 6A–F, 7A–D). From stage IV onwards, the continuous belt disappears and only the rectangular domains are visible along the antero-posterior body axis. Similar to the *engrailed* stripes, the *cubitus interruptus* domains do not show clear expression boundaries but rather a graded signal towards the margins of each domain (Figures 6D, F, 7D). The largest unitary expression domain occurs in the cephalic lobes of the antennal segment, in which only two transverse dorsal regions lack expression (arrows in Figure 6A, C).

When the limbs arise, the expression of *cubitus interruptus* persists in the anterior region of each limb bud, where it is expressed in both ectoderm and mesoderm (Figure 6E). At advanced developmental stages, the continuous belt of *cubitus interruptus* expression is no longer evident, as it disintegrates completely into separate segmental domains (Figures 6F, 7D). Similar to *engrailed* stripes (cf. Figure 4C), each *cubitus interruptus* domain consists of a lateral and a median portion, each of which has a different fate further in development, while the lateral portion (including the limb bud; arrowheads in Figure 7D) persists until late in development, the median portion (including the anlage of ventral/preventral organs; asterisks in Figure 7D) disappears earlier, following the anterior-to-posterior progression.

**Figure 8:**
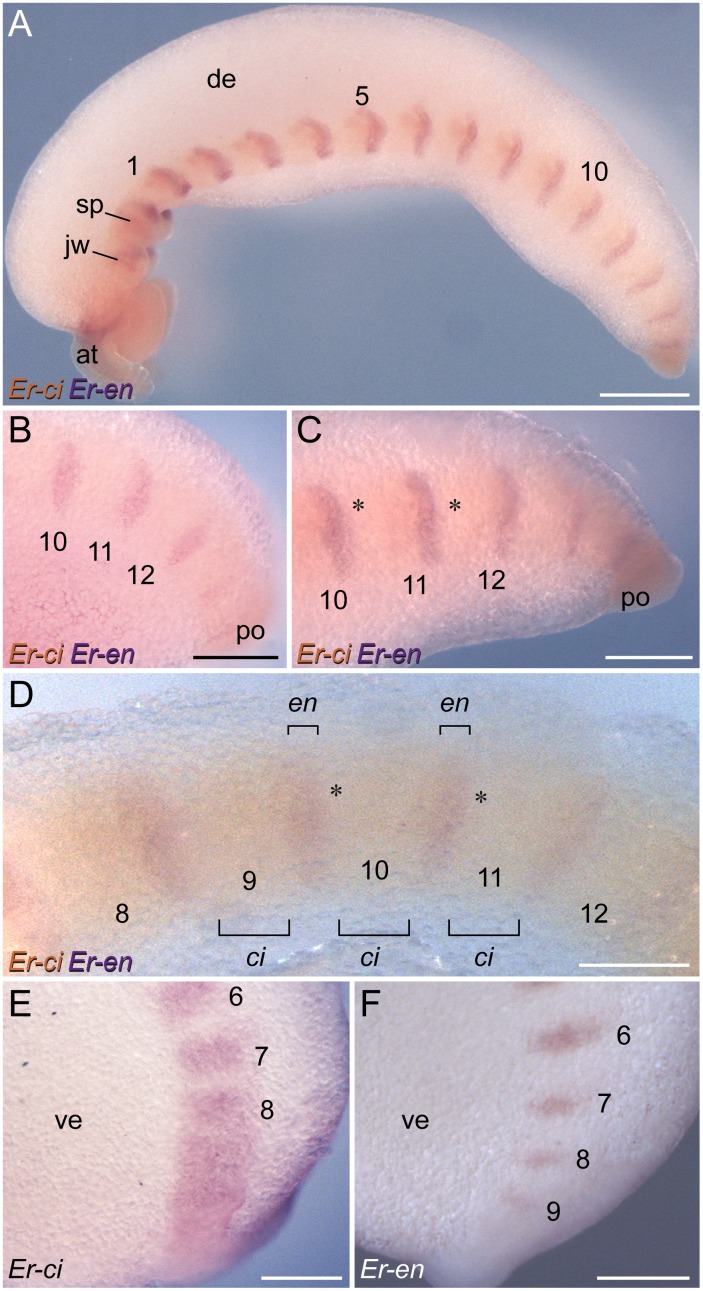
Localisation of *engrailed* and *cubitus interruptus* expression in embryos of *E. rowelli*. Labelling for *engrailed* is illustrated in purple and for *cubitus interruptus* in orange in A–D. Embryos in lateral view. Anterior is left and dorsal is up in all images. Leg-bearing segments are numbered. (A) Overview of a stage IV embryo. (B) Posterior end of a stage III embryo. Note that no gaps are evident between the *engrailed* and *cubitus interruptus* domains. Note also that there might be a co-expression of the two genes at least in cells located within the posterior-most *engrailed* stripes, as there is a continuous belt of *cubitus interruptus* expression in this body region at that stage (cf. E and F; see also Figures 6F, 7B). (C) Posterior end of a stage IV embryo. Asterisks indicate gaps between subsequent *engrailed* and *cubitus interruptus* domains. (D) Through-light micrograph showing subsequent domains of *engrailed* and *cubitus interruptus* in a stage IV embryo. Note gaps between the *engrailed* and *cubitus interruptus* domains (asterisks). (E) Expression of *cubitus interruptus* at the posterior end of a stage II embryo. (F) Expression of *engrailed* at the posterior end of a stage II embryo. Abbreviations: *at*, antenna; *ci*, *cubitus interruptus* domains; *de*, dorsal extra-embryonic tissue; *en*, *engrailed* domains; *jw*, jaw; *po*, proctodaeum; *sp*, slime papilla; *ve*, ventral extra-embryonic tissue. Scale bars: 500 µm (A), 250 µm (B, C, E, F), 200 µm (D).

To analyse the spatial relationship between the expression patterns of *cubitus interruptus* and *engrailed*, we conducted additional single and double *in situ* hybridization experiments (Figure 8A–F). Due to a persisting continuous belt of *cubitus interruptus* expression at the posterior end of the embryo (cf. Figure 6A), the domains of *engrailed* and *cubitus interruptus* overlap initially in this body region (Figure 8B, E, F). After the subdivision of this belt into separate, segmentally repeated domains, gaps occur between the *cubitus interruptus* and *engrailed* domains (Figure 8C, D). This is in line with the results of our single *in situ* hybridization experiments, which revealed gaps between adjacent *cubitus interruptus* domains that are wider than each *engrailed* stripe (cf. Figures 3A, 6A).

### Dynamic patterns of *wingless* expression during development in *E. rowelli*

**Figure 9:**
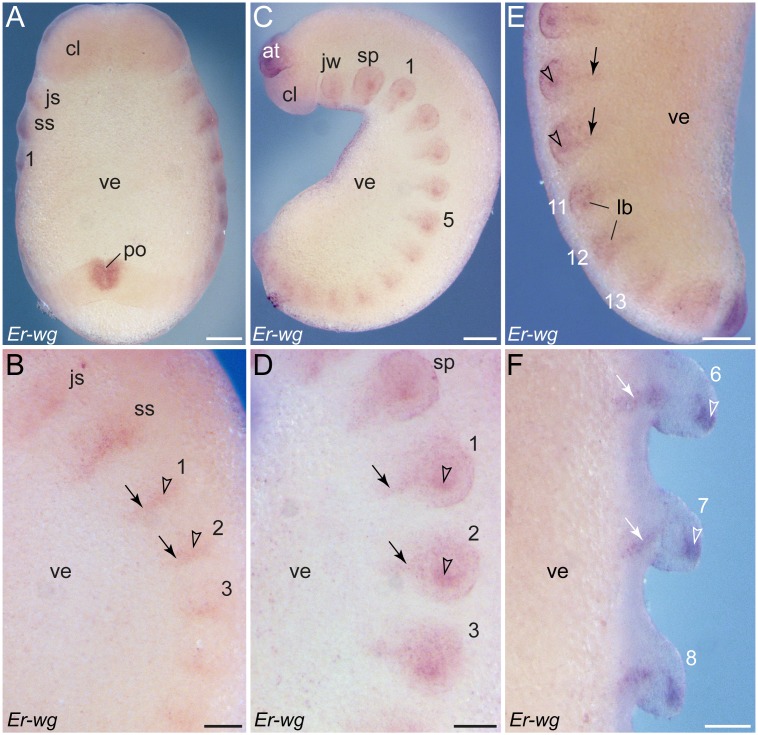
Expression of *wingless* in embryos of *E. rowelli*. Anterior is up in all images. Leg-bearing segments and corresponding limbs are numbered. (A) Stage II embryo in ventral view. Note that *wingless* is expressed in segmentally repeated stripes in the anlagen of limbs and in areas in which the limb buds arise. Note also *wingless* expression in the cephalic lobes and the proctodaeum. (B) Detail of *wingless* expression in the same embryo as in A in lateral view. Note that the expression first appears in the limb buds (arrowheads), followed by stripe-shaped domains in the ventral ectoderm (arrows). (C) Stage III embryo in lateral view. Note a weak and ubiquitous expression of *wingless* in the cephalic lobes and a strong expression in the anlagen of antennae. (D) Detail of *wingless* expression of the same embryo as in D in lateral view. Arrows point to appearing stripes along the ventral body surface, whereas arrowheads denote the spot-shaped domains in the distal portion of each developing limb. (E) Posterior end of a stage IV embryo in ventro-lateral view. As in the stage II and III embryos, the expression first appears in the limb buds (arrowheads), followed by stripe-shaped domains in the ventral ectoderm (arrows). (F) Detail of a stage IV embryo in ventral view. Note that the stripes in the ventral ectoderm are separated by non-expressing cells from the spot-shaped domains in the distal limb portions. Abbreviations: *at*, developing antenna; *cl*, cephalic lobe; *js*, jaw segment; *jw*, embryonic jaw; *lb*, limb buds; *po*, proctodaeum; *sp*, embryonic slime papilla; *ss*, slime papilla segment; *ve*, ventral extra-embryonic tissue. Scale bars: 250 µm (A, C), 125 µm (B, D), 200 µm (E), 100 µm (F).

Similar to *engrailed*, *wingless* is expressed in a reiterated pattern along the germ band (Figure 9A–F). However, in contrast to *engrailed*, this gene is initially expressed in the early anlagen of limbs (arrowheads in Figure 9B) and each segmental domain extends subsequently in a stripe-like fashion to the ventral ectoderm (arrows in Figure 9B), following the anterior-to-posterior progression in development. Each stripe demarcates the middle of each limb basis, from which it runs further medially (Figure 9C–F). When the limb buds elongate, the spot-shaped domains become more prominent at the tip of each developing limb, including the slime papillae and the jaws (Figure 9B–D). As development proceeds, the initial unitary domain associated with each limb is subdivided into two separate domains: a spot-shaped domain at the tip of each limb, and a stripe-shaped domain in the ventral ectoderm, including the limb basis (Figure 9D–F). In addition to these domains along the body, a ubiquitous expression of *wingless* occurs in the cephalic lobes and the antennae and a conspicuous ring-shaped domain is seen around the proctodaeum (Figure 9A, C, E).

### Posterior expression of *hedgehog* in each segment in embryos of *E. rowelli*

**Figure 10:**
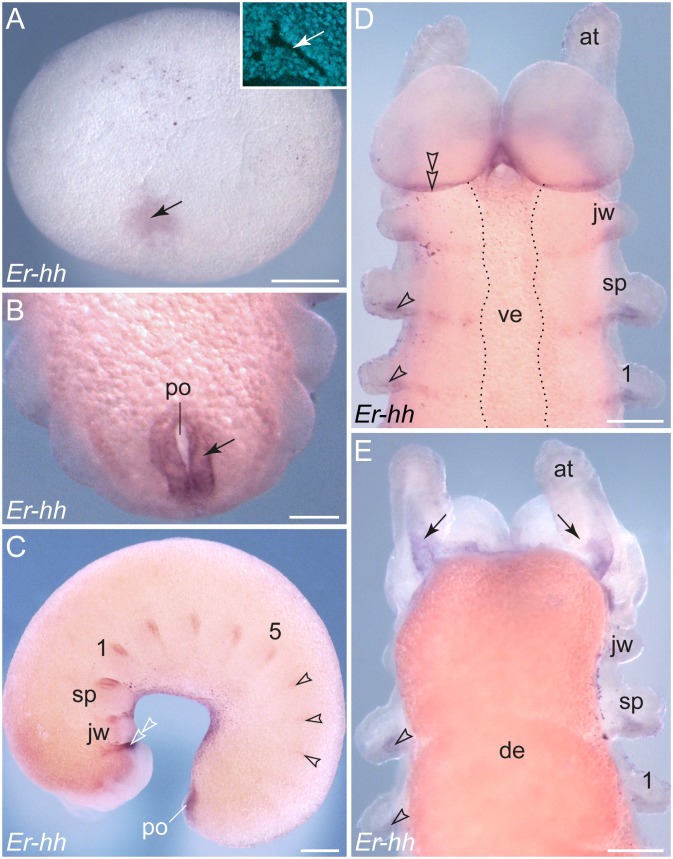
Expression of *hedgehog* in embryos of *E. rowelli*. Leg-bearing segments and corresponding limbs are numbered. Arrowheads indicate segmentally repeated stripes in the posterior portion of each developing limb. (A) Stage I embryo. Inset in the upper right corner shows a fluorescent micrograph (DNA labelling) of the blastoporal area from the same embryo. Arrows point to the position of the blastopore, which is surrounded by *hedgehog* expressing cells. (B) Posterior end of a stage IV embryo. Arrow indicates the expression around the proctodaeum. (C) Stage III embryo in lateral view showing repeated stripes of expression along the body. Arrowheads point to the emerging *hedgehog* domains in the posterior portion of each developing limb in the posterior half of the embryo. Note an elongated domain at the posterior border of the cephalic lobes (double-arrowhead). (D) Anterior end of a stage IV embryo in ventral view. Double-arrowhead points to the elongated domain at the posterior border of the cephalic lobes. Dotted line indicates the border between the ectoderm and the ventral extra-embryonic tissue. (E) Dorsal view of the same embryo as in D. Arrows point to the expression in the mesoderm at the bases of the developing antennae. Abbreviations: *at*, developing antenna; *de*, dorsal extra-embryonic tissue; *jw*, embryonic jaw; *po*, proctodaeum; *sp*, embryonic slime papilla; *ve*, ventral extra-embryonic tissue. Scale bars: 500 µm (A), 100 µm (B), 250 µm (C), 200 µm (D, E).

The earliest detectable expression of *hedgehog* occurs around the early elongating blastopore (Figure 10A). Later in development, a ring-shaped domain is still seen around the proctodaeum (Figure 10B, C), thus resembling the expression pattern of *wingless* in the same region (cf. Figure 9A, E). In addition to this posterior domain, stripes of *hedgehog* expression emerge sequentially along the embryo, following the anterior-to-posterior progression in development (Figure 10C). This pattern is similar to the expression of *engrailed*, but the *hedgehog* stripes are thinner and shorter than the *engrailed* stripes and are expressed later, first appearing in each developing limb bud (Figure 10C).

**Figure 11:**
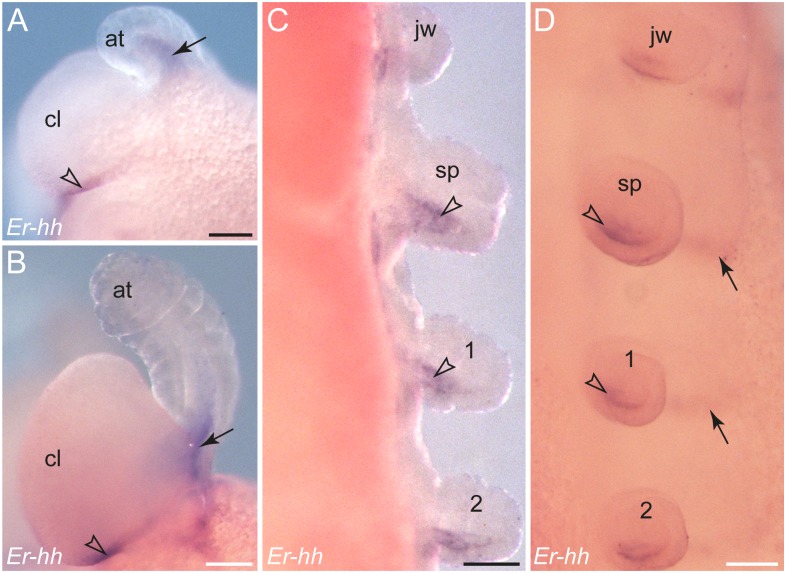
Details of *hedgehog* expression in embryos of *E. rowelli*. Leg-bearing segments and corresponding limbs are numbered. (A, B) Anterior ends of a stage III and a stage IV embryo in lateral view. Arrows point to the expression in the mesoderm of the developing antennae, whereas arrowheads indicate the expression at the posterior border of the cephalic lobe. (C, D) Details of limbs in a stage IV embryo in dorsal (in C) and ventro-lateral views (in D). Arrowheads indicate the expression in the mesoderm of each developing limb, although expression is also evident in the ectoderm of limb anlagen. Arrows point to stripes of expression in the ventral ectoderm. Abbreviations: *at*, antenna; *cl*, cephalic lobe; *jw*, jaw segment; *po*, proctodaeum; *sp*, slime papilla. Scale bars: 100 µm (A–D).

Within the limb buds, *hedgehog* is expressed posteriorly in both ectoderm and mesoderm as a graded signal; only in the antennae is this gene expressed dorsally (Figures 10C–E, 11A–D). During further development, the *hedgehog* stripes extend medially in the ventral ectoderm (Figure 10D, 11D). These median domains are located posterior to the corresponding *wingless* domains (see Figure S4). The most prominent stripe of *hedgehog* expression demarcates the posterior boundary of the cephalic lobes in the antennal segment (Figures 10C, D, 11A, B, D).

## Discussion

### Segments, rather than parasegments, are the initial metameric units in the onychophoran embryo

Parasegments are believed to be the initial metameric compartments of the arthropod embryo [3], [7], [13], [14], . They arise early in development and are recognised by several features, including (i) the juxtaposed pattern of *wingless* and *engrailed/hedgehog* expression (via an autoregulatory interaction of these genes, which was initially demonstrated in embryos of *Drosophila melanogaster* [7], [15], [83], [84]); (ii) conspicuous expression patterns of some Hox genes, obeying the parasegmental boundaries [7], [11], [16], [83], [85]; and (iii) cell lineages that at least in crustaceans are restricted to the genealogical units corresponding to parasegments [86]–[90]. Thus, if present, parasegments should be recognisable in the onychophoran embryo based on these criteria.

**Figure 12:**
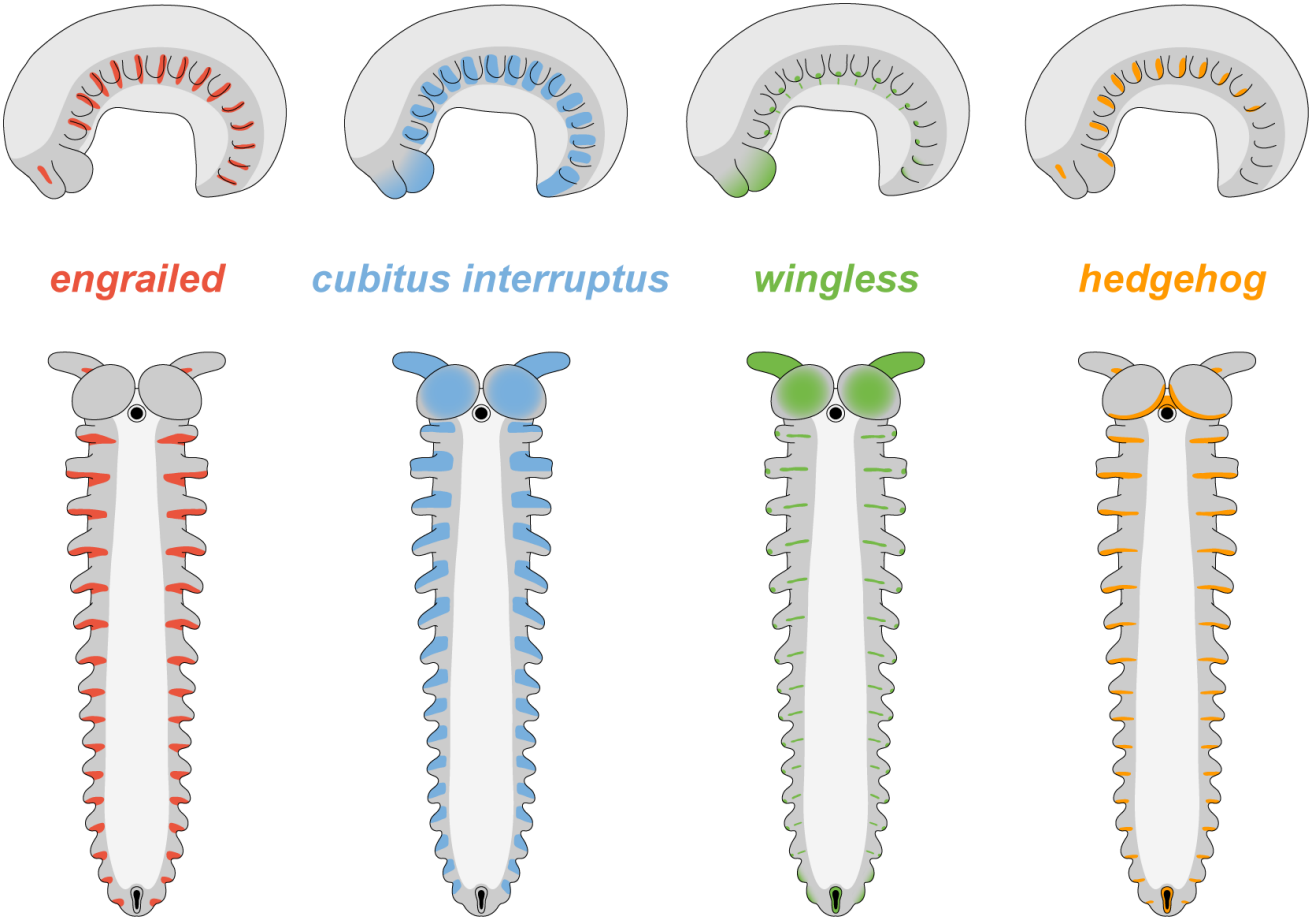
Diagrams of *engrailed*, *cubitus interruptus*, *wingless* and *hedgehog* expression in embryos of the onychophoran *E. rowelli*. The upper row illustrates stage III embryos in lateral view, whereas the lower row shows stage IV embryos in ventral and lateral views, respectively. Note the segmentally repeated patterns of expression of all four “segment polarity genes” and their specific order within each segment (see text for further details).

**Figure 13:**
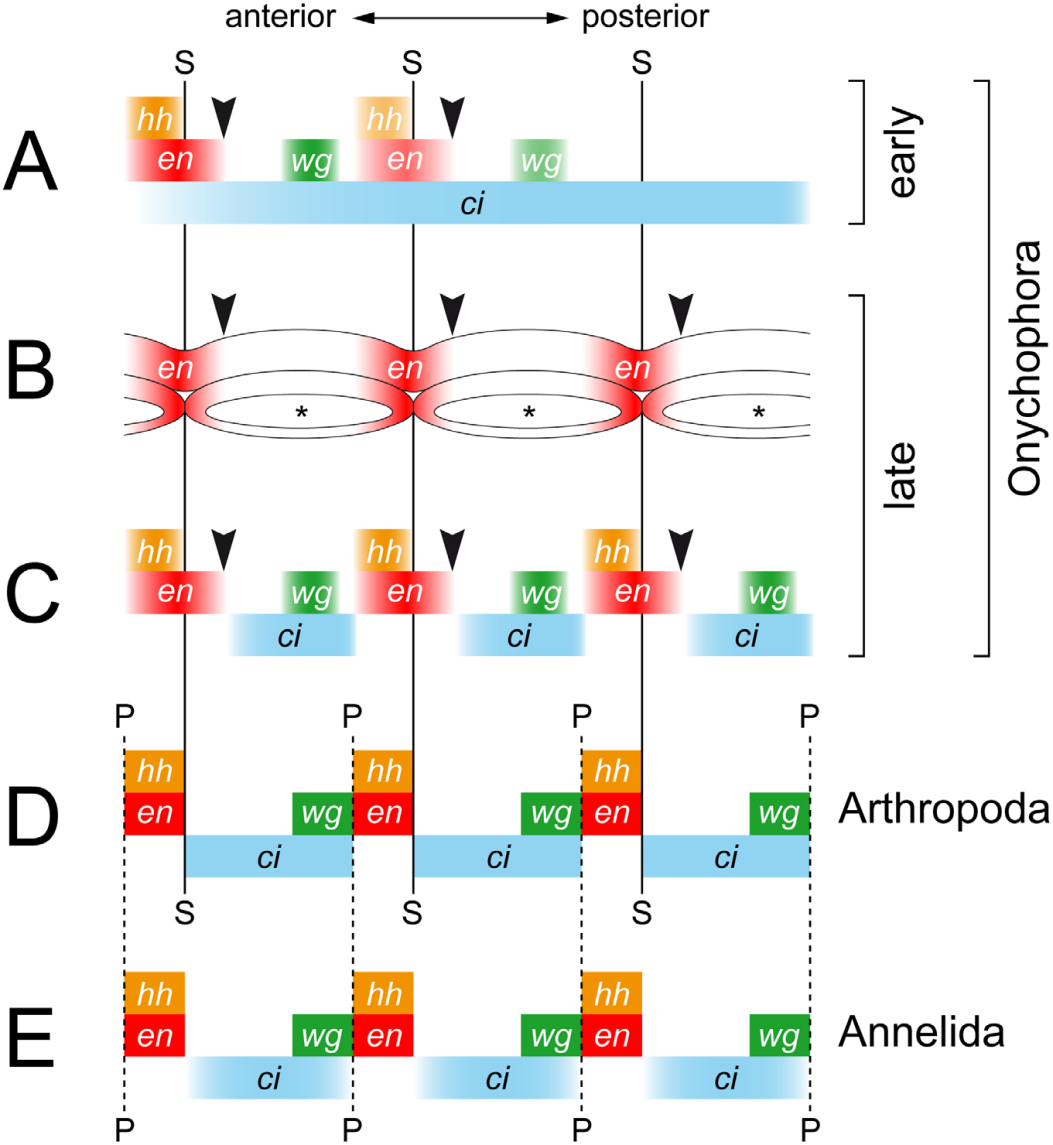
Diagrams comparing the expression patterns of homologs of *engrailed*, *cubitus interruptus*, *wingless* and *hedgehog* along the body in onychophorans, arthropods and annelids. Vertical lines demarcate the position of segmental (solid lines) and parasegmental boundaries (hatched lines) only for taxa, in which they are identifiable morphologically. Black arrowheads indicate the position of expected, albeit non-existent, segmental boundaries in Onychophora, which is in contrast to arthropods. (A) Diagram of expression in embryos of the onychophoran *Euperipatoides rowelli* at an early developmental stage. Note the continuous belt of *cubitus interruptus* expression. (B) Diagram of expression of *engrailed* in the ectoderm and the mesodermal somites in the onychophoran *Euperipatoides kanangrensis* (based on previous data [29]). Asterisks indicate coelomic cavities. Note that the expression pattern does not correspond to the segmental boundaries and that each somite shows an anterior and a posterior expression domain. (C) Diagram of expression in embryos of the onychophoran *E. rowelli* at a late developmental stage. Note the graded expression patterns that do not form distinct boundaries (cf. ref. [29]). (D) Diagram of expression in the fruit fly *Drosophila melanogaster*, modified from [24]. (E) Diagram of expression in the annelid *Platynereis dumerilii*, modified from [24]. Abbreviations: *ci*, *cubitus interruptus*; *en*, *engrailed*; *hh*, *hedgehog*; *P*, parasegmental boundary; *S*, segmental boundary (in onychophorans only seen during embryogenesis); *wg*, *wingless*.

Our data from *E. rowelli*, as well as those from the closely related species *E. kanangrensis* [29], [39], show that *engrailed*, *cubitus interruptus*, *wingless* and *hedgehog* are all expressed in a reiterated pattern in the onychophoran embryo (Figure 12). The expression occurs in repeated sets along the body, the relative order of which corresponds to that in arthropods; *cubitus interruptus* and *wingless* are expressed anterior to the *engrailed* and *hedgehog* domains (Figure 13A–D) [16], [17], [22], [83], [91], [92]. These data are in line with the assumption of an autoregulatory interaction of these genes [7], [13], [14], although the graded expression pattern in the trunk lacks boundaries in onychophorans. Nonetheless, one could argue that a distinct boundary might still exist at the post-transcriptional level [29], which would correspond to the parasegmental boundary of arthropods (Figure 13D).

Another piece of evidence for parasegments in arthropods comes from the anterior expression borders of the posterior Hox genes, which typically correspond to the parasegmental boundaries, including *Antennapedia*, *Ultrabithorax*, *abdominal-A*, *Abdominal-B* in chelicerates, *fushi tarazu*, *Abdominal-B* in myriapods, *proboscipedia* in crustaceans, and *Sex combs reduced*, *Antennapedia*, *abdominal-A* and *Abdominal-B* in hexapods [7], [11], [14], [16], [83], [85], [93]. However, the expression patterns of Hox genes in the onychophoran *E. kanangrensis* and localisation of the Ultrabithorax and abdominal-A proteins in *Acanthokara kaputensis* mostly revealed segmental rather than parasegmental patterns, which is different from the situation in arthropods [3], [93]. The anterior expression borders of seven of the ten Hox genes in *E. kanangrensis* clearly do not lie between the *wingless* and *engrailed/hedgehog* domains but instead correspond to the segmental furrows [94], [95]. In contrast to this, the anterior expression borders of *Hox3*, *Sex combs reduced* and *fushi tarazu* have been interpreted as being positioned “in the middle” [95] of the corresponding segments. However, the position of the anterior expression borders of these three genes in relation to the *engrailed* and *wingless* domains still needs to be analysed to clarify whether or not these genes are expressed in a “parasegmental” pattern.

In addition to gene expression studies, cell lineage analyses revealed a correlation between the clonal and parasegmental boundaries in crustaceans [86−90], . The progeny of cells on either side of the parasegmental boundary remain separated, suggesting that parasegments are real genealogical units [6], [89], [96]. These units are reflected in the parasegmental organisation of the ventral nerve cord in various arthropods, including the fruit fly *Drosophila melanogaster* [6], [20], [21]. During *Drosophila* development, each neuromere originates from cells located between two consecutive parasegmental furrows [21] and eventually gives rise to a ganglion. Therefore, due to their out-of-register nature, each resulting ganglion is shifted anteriorly once the embryo is re-segmented [6], [7]. This shifted arrangement of ganglia is a common feature of all arthropod groups, especially in body regions that have retained the ancestral, ladder-like organisation of the ventral nerve cord with separate, metameric ganglia [6]).

In contrast to arthropods, the onychophoran nerve cord displays no such metameric ganglia (cf. Figure 1I, J; [31], [97], [98]). Unfortunately, cell lineage analyses, which would unveil the boundaries of putative genealogical units and their relationship to the nervous system, are currently unfeasible in Onychophora. However, neuronal tracing of leg nerves revealed no anterior shift in the arrangement of motor neurons in the nerve cords of the onychophoran *E. rowelli* [37], which contrasts with the anteriorly shifted ganglia and motor neurons in arthropods [6]. Thus, there is currently no neuroanatomical indication of parasegments in adult onychophorans.

In addition to the ganglia of arthropods, the parasegmental boundaries are also manifested morphologically as transverse grooves in the embryonic ectoderm of some chelicerate [16], crustacean [19] and insect species [14], [20], [21], although embryonic grooves are not always recognisable e.g., in decapod crustaceans [99]. During re-segmentation of the embryo, each ectodermal groove situated in a parasegmental position (anterior to the *engrailed* domain) is replaced by a new groove in a segmental position (posterior to the *engrailed* domain) (Figure 13D; [16], [19], [100], [101]). This contrasts with our findings, which instead show that the transverse furrow in *E. rowelli* embryos does not change its position during development, thus providing no indication for a re-segmentation of the embryo. Moreover, the furrow is not located anterior to the *engrailed* domain but rather corresponds to the segmental border between adjacent somites (Figure 13B). Therefore, in contrast to arthropods, neither the embryonic ectoderm nor the organisation of the adult nervous system provides evidence for a morphological manifestation of parasegments in Onychophora.

Despite the lack of morphological evidence, we cannot exclude that parasegments, as defined by the autoregulatory interaction of “segment polarity genes” [7], [14], [16], [18], might still exist in Onychophora, at least at the post-transcriptional level [29]. However, the present and previous data from the onychophoran embryo clearly show that *wingless*, *engrailed* and *hedgehog* are expressed after the segmental boundaries have been established [29], [39] (Figure 13A–C). Therefore, the parasegments cannot be the initial metameric units in the onychophoran embryo, because they are preceded by segments, which are recognisable by segmental furrows, dorsal indentations of the germ band, and metameric somites. This clearly contrasts with the situation in arthropods, in which parasegments rather than segments are the initial metameric units of the embryo [3], [7], [13], [14], [16], [18].

### “Segment polarity genes” are not involved in segment formation in Onychophora

Our data from *E. rowelli* show that the timing of expression of *engrailed*, *cubitus interruptus*, *wingless* and *hedgehog* is entirely different from that in arthropods (Figure 13A–D). An initial belt of expression of *cubitus interruptus* occurs at the posterior end in *E. rowelli*, but this gene is unlikely to be involved in segment formation because this posterior belt dissociates into metameric domains only after the segmental furrows have formed. Likewise, the segmental domains of the three remaining genes occur after the establishment of the segmental furrows, suggesting that these genes play no role in segment formation, in contrast to what occurs in arthropods [17], [40], [102]. Additionally, our data confirm that each *engrailed* domain extends beyond the segmental furrow in the ectoderm of the onychophoran embryo [29]. The same holds true for the mesoderm, in which *engrailed* is expressed beyond the border of adjacent somites (Figure 13B; [29]). Thus, there is no clear spatial relationship between the formation of segmental boundaries and the expression of *engrailed* in Onychophora (arrowheads in Figure 13B, C).

Although the anterior-to-posterior order of expression of “segment polarity genes” might be conserved in arthropods and onychophorans [39], the lack of a spatial and temporal relationship between the expression domains and the segmental boundaries speaks against the involvement of these genes in segment formation in Onychophora. A similar lack of correlation has been demonstrated recently for most pair rule genes in the onychophoran *E. kanangrensis* [39], indicating that there might be an additional, early segment patterning mechanism in the onychophoran embryo. Identifying this underlying mechanism would be key to understanding the evolution of segmentation in Panarthropoda (Onychophora + Tardigrada + Arthropoda).

### Concerted patterning of segmental structures during onychophoran development

Our data further revealed spatiotemporal differences in the expression patterns between the median and lateral portions of the germ band in *E. rowelli* embryo for all four “segment polarity genes” studied. These differences are more evident in advanced developmental stages, in which the lateral domains are associated with the developing limbs, whereas the median domains correspond to the anlagen of the ventral and preventral organs [30], [42], [56]. These findings correspond to the previously published data on *engrailed* and *wingless* expression in *E. kanangrensis*, where the anlagen of the ventral and preventral organs (cf. refs [30], [42]) were instead interpreted as the developing nerve cords [29].

A similar correlation between the expression patterns of “segmentation genes” and organogenesis was observed recently in *E. kanangrensis*, in which the “pair rule gene” *odd-skipped* is expressed in the segmental anlagen of nephridia [39]. These findings imply that the observed expression patterns are associated with the individual segmental structures [8], [10], [46] rather than with a segment as a holistic unit [43]–[45]. This might explain why the sets of substructures comprising a ‘segment’ [9], [45] differ between onychophorans and arthropods. Therefore, we suggest that the segmental structures and organs that do not have any homologues in arthropods (and other animals; cf. Figure 1A–L) might have evolved in the onychophoran lineage, after the segmentation of the body and the corresponding patterning mechanisms responsible for a concerted positioning of such structures were already present.

## Conclusions

Based on the relative position of segmental and parasegmental boundaries and similarities in the expression patterns of the segment polarity genes (Figure 13D, E), the embryonic parasegments of arthropods have been homologised with the adult segments of annelids [24], [25]. This implies that the last common ancestor of protostomes possessed parasegments, whereas definitive segments evolved in arthropods [24], [25], [103]. According to this hypothesis, one would expect that parasegments also occur in one of the closest arthropod relatives, the Onychophora [24], [29]. However, our data suggest that despite the conserved anterior-to-posterior order of expression of the segment polarity genes, the mechanisms of segment formation might be fundamentally different in Onychophora. Although gene expression studies have provided useful insights into segment formation and body patterning in various animals, the complexity and plasticity of the mechanisms involved are still poorly understood, especially in non-model organisms, such as onychophorans. The fundamental differences in segment patterning in Onychophora revealed in this and previous studies [29], [39], [104] suggest that it might be premature to speculate on the common origin of segmentation in distantly related animal groups, such as annelids and arthropods [24], [25], [105]. We caution that the similarities at the transcriptional level might be superficial due to an independent recruitment of the same canonical signalling pathways, most of which are certainly older than the origin of segmentation [106]–[108].

## Supporting Information

Figure S1 **Sequence alignments for** ***cubitus interruptus*** **,** ***engrailed*** **,** ***hedgehog*** **and** ***wingless*** **.**
(PDF)

Figure S2 **Early development of the embryonic furrows in embryos of** ***E. rowelli*** **.** Confocal micrographs of embryos, double-labelled with the DNA marker Bisbenzimide (A, C) and the f-actin marker phalloidin-rhodamine (B, D). The images in A and C are from the same embryos as in Figure 2C and D. (A, B) Posterior end of a stage II embryo. Note that the segmental furrows have not been formed yet. (C, D) Posterior end of a stage III embryo. Arrows point to the segmental furrows in the ectoderm (in C) and between the mesodermal somites ( = coelomic cavities, marked by asterisks in D). Abbreviations: *po*, proctodaeum; *ve*, ventral extra-embryonic tissue. Scale bars: 100 µm (A–D).
(TIF)

Figure S3 **Cladograms based on phylogenetic analyses of** ***engrailed*** **,** ***cubitus interruptus*** **,** ***hedgehog*** **and** ***wingless*** **sequences using RAxML.** Numbers at nodes are maximum likelihood bootstrap values (100 replicates). Sequences of the onychophoran *E. rowelli* are highlighted in bold/red. For the analyses of *engrailed* and *hedgehog* phylogenies, *Platynereis dumerilii* was used as an outgroup. For the analysis of *cubitus interruptus* phylogeny, *Achaearanea tepidariorum* was selected as an outgroup. For the analysis of *wingless*, we set up an alignment of several *Wnt1* and *Wnt6* sequences from [109] and used *Wnt6* from different taxa as an outgroup (note that the sequences from the onychophorans *E. rowelli* and *E. kanangrensis* cluster together within the *Wnt1* clade).
(TIF)

Figure S4 **Expression of** ***wingless*** **and** ***hedgehog*** **in embryos of** ***E. rowelli*** **.** Leg segments of stage IV embryos in ventral view. Note that the *hedgehog* stripes are located posterior to the corresponding *wingless* domains. Abbreviation: *le*, legs. Scale bars: 100 µm (A, B).
(TIF)

Table S1 **List of species and genes with corresponding accession numbers used for phylogenetic analyses.**
(PDF)
