# Supplementary material for: Controversies Surrounding Segments and Parasegments in Onychophora: Insights from the Expression Patterns of Four “Segment Polarity Genes” in the Peripatopsid Euperipatoides rowelli
Source: PLoS One. 2014 Dec 3;9(12):e114383. doi: 10.1371/journal.pone.0114383 (PMC4255022; doi:10.1371/journal.pone.0114383)

cubitus interruptus

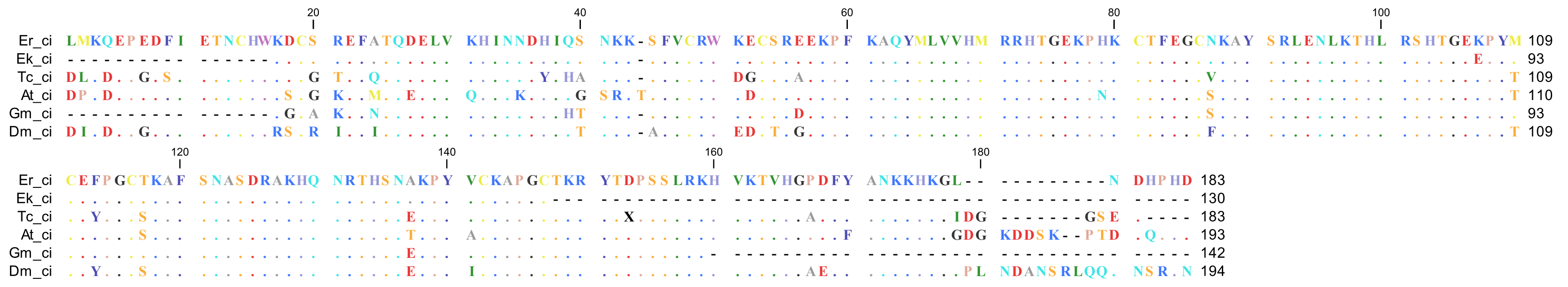

engrailed

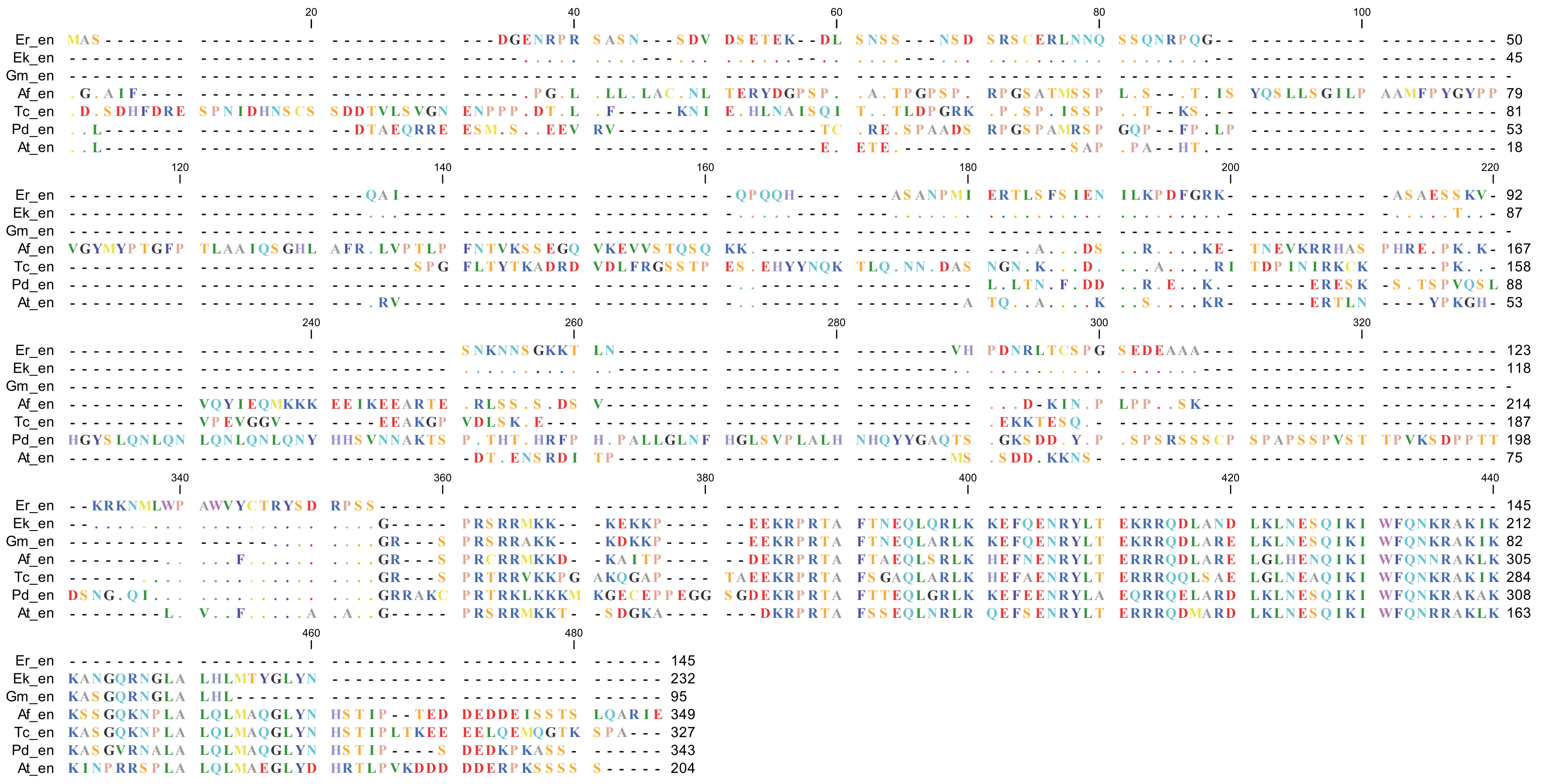

hedgehog

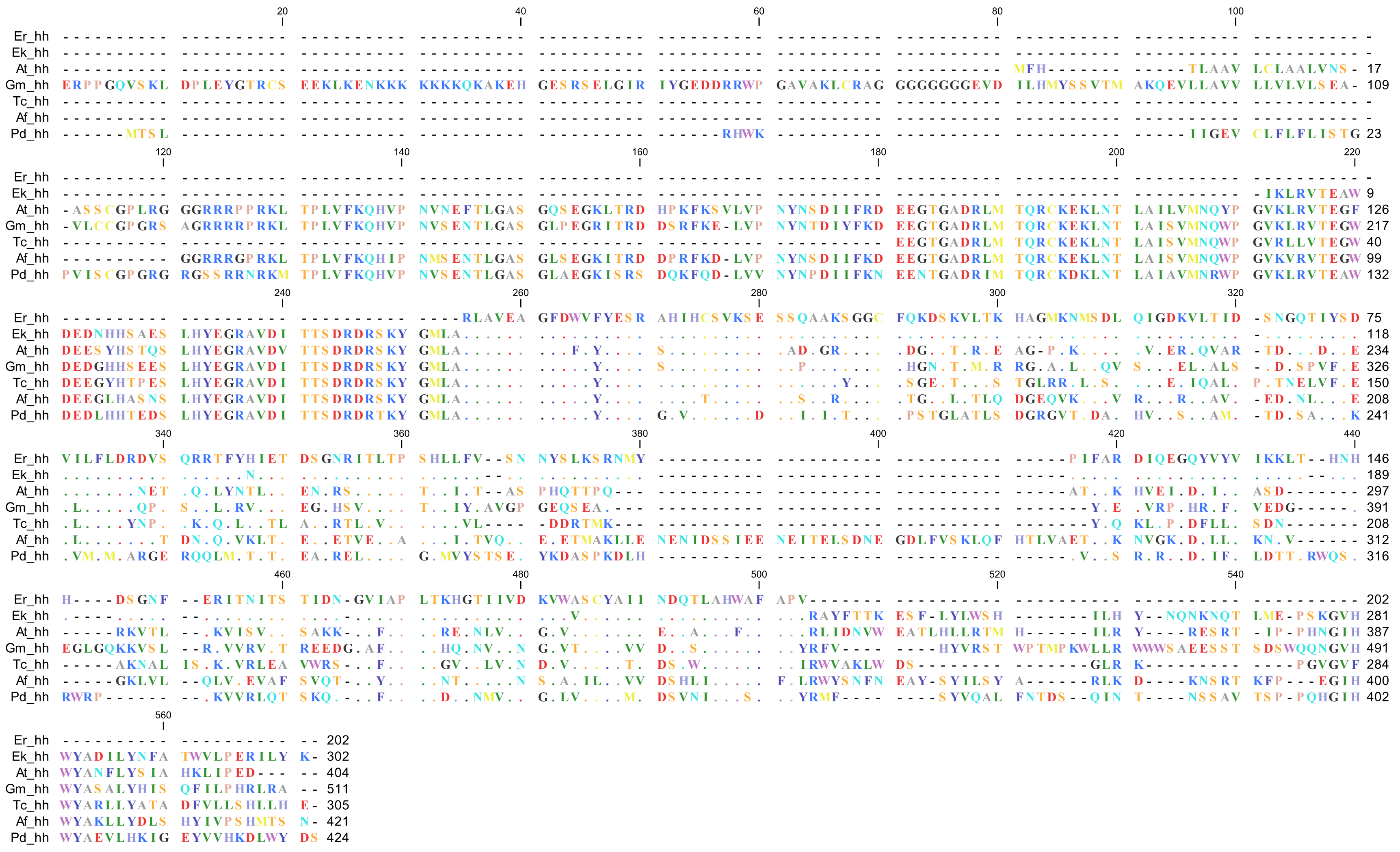

wingless

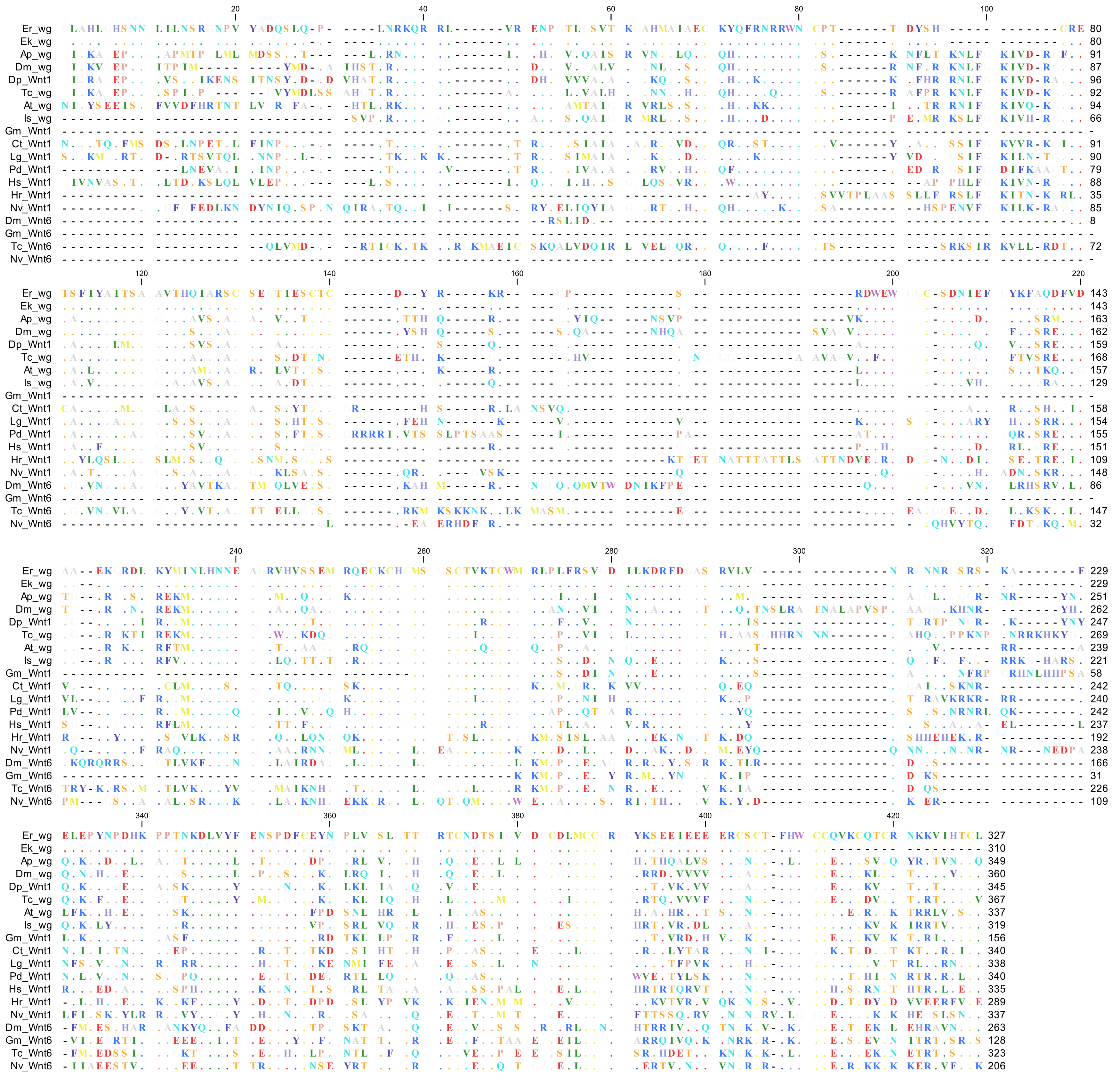

Supplement: Figure S1 — Sequence alignments for cubitus interruptus , engrailed , hedgehog and wingless . (PDF) [file pone.0114383.s001.pdf]
