# Supplementary figures and images for: Controversies Surrounding Segments and Parasegments in Onychophora: Insights from the Expression Patterns of Four “Segment Polarity Genes” in the Peripatopsid Euperipatoides rowelli
Source: PLoS One. 2014 Dec 3;9(12):e114383. doi: 10.1371/journal.pone.0114383 (PMC4255022; doi:10.1371/journal.pone.0114383)

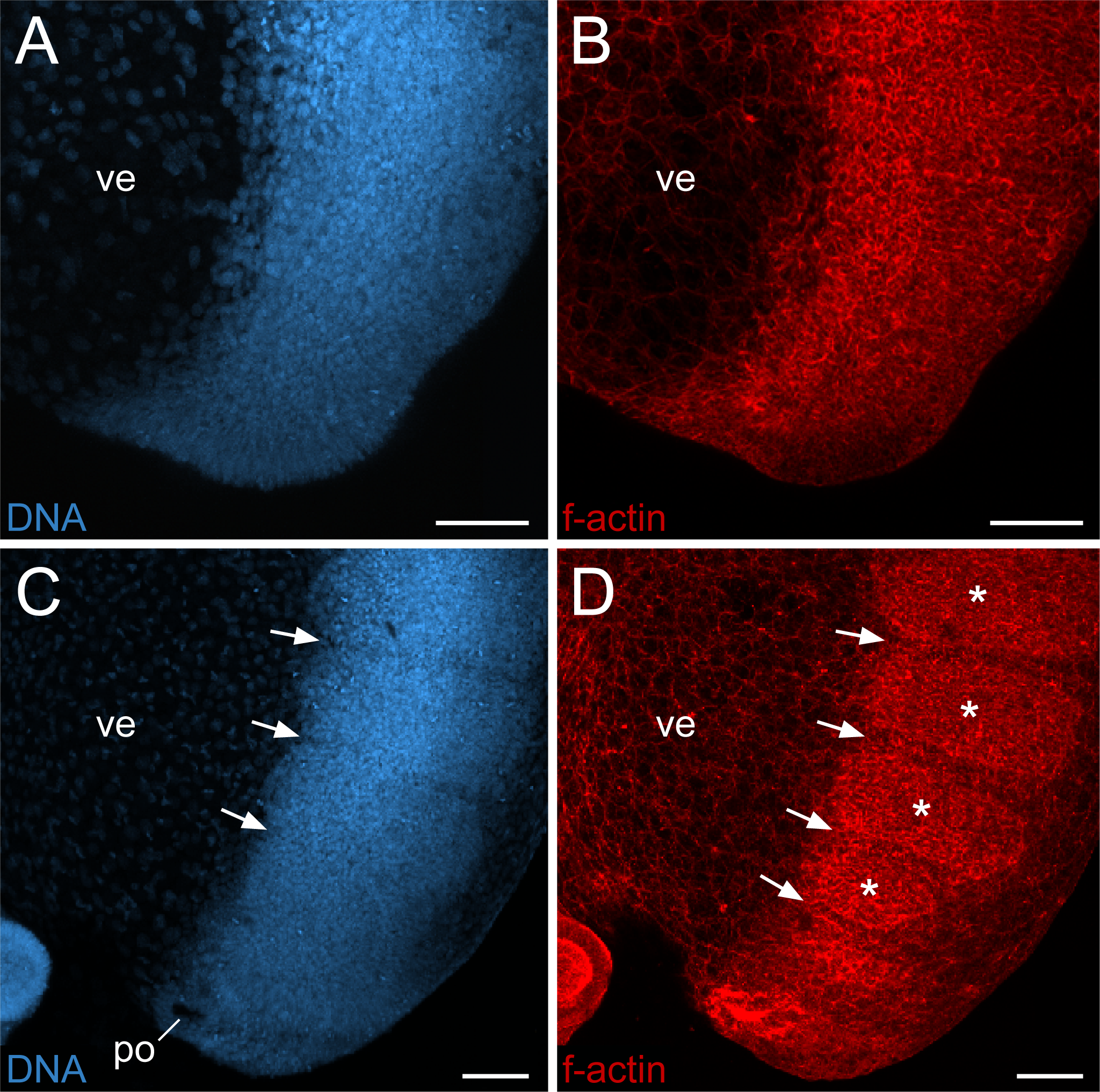

Supplement: Figure S2 — Early development of the embryonic furrows in embryos of E. rowelli . Confocal micrographs of embryos, double-labelled with the DNA marker Bisbenzimide (A, C) and the f-actin marker phalloidin-rhodamine (B, D). The images in A and C are from the same embryos as in Figure 2C and D. (A, B) Posterior end of a stage II embryo. Note that the segmental furrows have not been formed yet. (C, D) Posterior end of a stage III embryo. Arrows point to the segmental furrows in the ectoderm (in C) and between the mesodermal somites ( = coelomic cavities, marked by asterisks in D). Abbreviations: po, proctodaeum; ve, ventral extra-embryonic tissue. Scale bars: 100 µm (A–D). (TIF) [file pone.0114383.s002.tif]

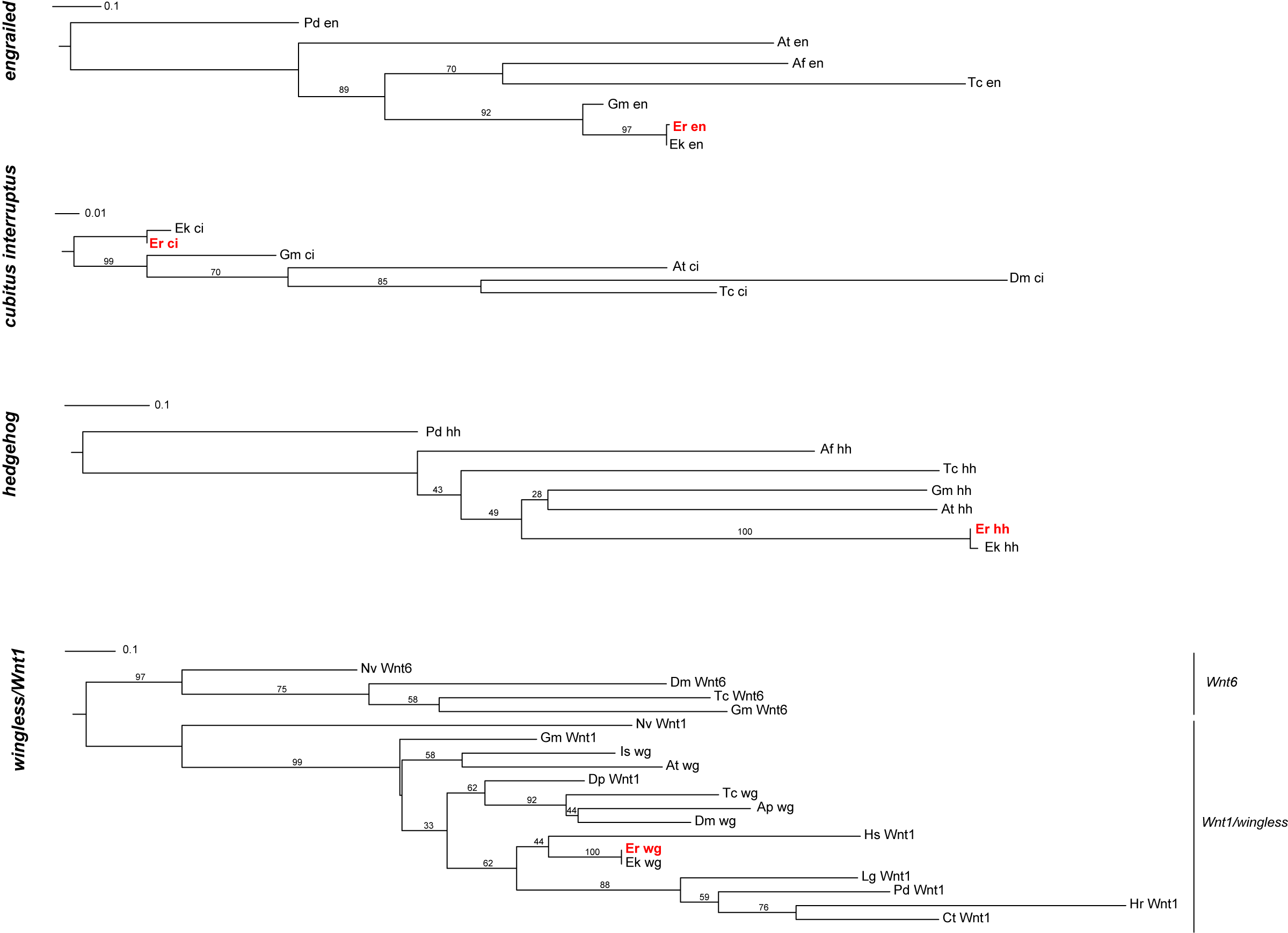

Supplement: Figure S3 — Cladograms based on phylogenetic analyses of engrailed , cubitus interruptus , hedgehog and wingless sequences using RAxML. Numbers at nodes are maximum likelihood bootstrap values (100 replicates). Sequences of the onychophoran E. rowelli are highlighted in bold/red. For the analyses of engrailed and hedgehog phylogenies, Platynereis dumerilii was used as an outgroup. For the analysis of cubitus interruptus phylogeny, Achaearanea tepidariorum was selected as an outgroup. For the analysis of wingless, we set up an alignment of several Wnt1 and Wnt6 sequences from [109] and used Wnt6 from different taxa as an outgroup (note that the sequences from the onychophorans E. rowelli and E. kanangrensis cluster together within the Wnt1 clade). (TIF) [file pone.0114383.s003.tif]

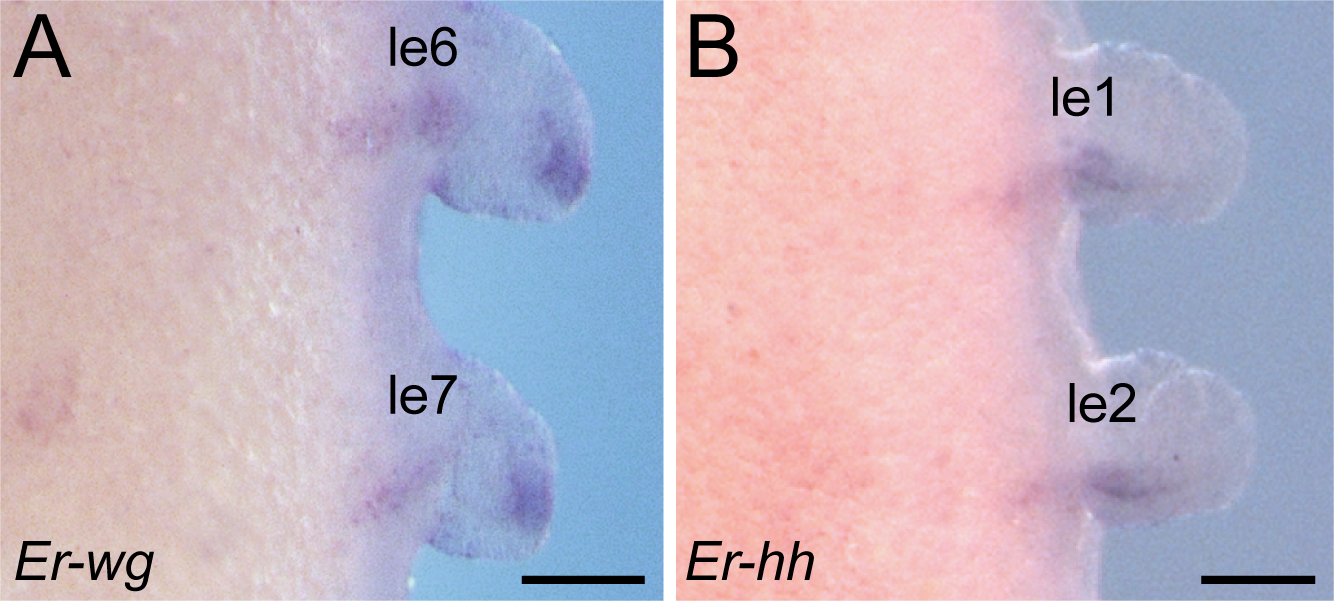

Supplement: Figure S4 — Expression of wingless and hedgehog in embryos of E. rowelli . Leg segments of stage IV embryos in ventral view. Note that the hedgehog stripes are located posterior to the corresponding wingless domains. Abbreviation: le, legs. Scale bars: 100 µm (A, B). (TIF) [file pone.0114383.s004.tif]
