# Supplementary material for: Controversies Surrounding Segments and Parasegments in Onychophora: Insights from the Expression Patterns of Four “Segment Polarity Genes” in the Peripatopsid Euperipatoides rowelli
Source: PLoS One. 2014 Dec 3;9(12):e114383. doi: 10.1371/journal.pone.0114383 (PMC4255022; doi:10.1371/journal.pone.0114383)
Supplement: Table S1 — List of species and genes with corresponding accession numbers used for phylogenetic analyses. (PDF) [file pone.0114383.s005.pdf]

List of genes, abbreviations and animal species with NCBI accession numbers used for phylogenetic analyses of *engrailed*, *cubitus interruptus*, *wingless* and *hedgehog*.

Gene	Abbreviation	Species	GenBank number
engrailed	Af en	*Artemia franciscana*	CAA50279
engrailed	At en	*Achaearanea tepidariorum*	BAD01489
engrailed	Ek en	*Euperipatoides kanangrensis*	ABY60731
engrailed	Gm en	*Glomeris marginata*	CAE83645
engrailed	Pd en	*Platynereis dumerilii*	CAE46753
engrailed	Tc en	*Tribolium castaneum*	NP001034511
cubitus interruptus	At ci	*Achaearanea tepidariorum*	BAK93298
cubitus interruptus	Dm ci	*Drosophila melanogaster*	NP524617
cubitus interruptus	Ek ci	*Euperipatoides kanangrensis*	CDF52130
cubitus interruptus	Gm ci	*Glomeris marginata*	CAE83647
cubitus interruptus	Tc ci	*Tribolium castaneum*	ACN4333
hedgehog	Af hh	*Artemia franciscana*	AAP38182
hedgehog	At hh	*Achaearanea tepidariorum*	BAD01490
hedgehog	Ek hh	*Euperipatoides kanangrensis*	CDF52128
hedgehog	Gm hh	*Glomeris marginata*	CAE83646
hedgehog	Pd hh	*Platynereis dumerilii*	ADK38669
hedgehog	Tc hh	*Tribolium castaneum*	ABF48588
wingless	Ek wg	*Euperipatoides kanangrensis*	ABY60732

Sequences were taken from the *Wnt* alignment of additional file 4 and accession numbers for Wnt genes are provided in additional file 1 of Janssen et al. 2010 [109].

Abbreviations of animal species: Ap, *Acyrthosiphon pisum*; At, *Achaearanea tepidariorum*; Ct, *Capitella teleta*; Dm, *Drosophila melanogaster*; Dp, *Daphnia pulex*; Ek, *Euperipatoides kanangrensis*; Er, *Euperipatoides rowelli*; Gm, *Glomeris marginata*; Hr, *Helobdella robusta*; Hs, *Homo sapiens*; Is, *Ixodes scapularis*; Lg, *Lottia gigantea*; Nv, *Nematostella vectensis*; Pd, *Platynereis dumeilii*; Tc, *Tribolium castaneum*.
